# Author Correction: Mapping chromatin accessibility and active regulatory elements reveals pathological mechanisms in human gliomas

**DOI:** 10.1038/s41467-021-27448-5

**Published:** 2021-12-06

**Authors:** Karolina Stępniak, Magdalena A. Machnicka, Jakub Mieczkowski, Anna Macioszek, Bartosz Wojtaś, Bartłomiej Gielniewski, Katarzyna Poleszak, Malgorzata Perycz, Sylwia K. Król, Rafał Guzik, Michał J. Dąbrowski, Michał Dramiński, Marta Jardanowska, Ilona Grabowicz, Agata Dziedzic, Hanna Kranas, Karolina Sienkiewicz, Klev Diamanti, Katarzyna Kotulska, Wiesława Grajkowska, Marcin Roszkowski, Tomasz Czernicki, Andrzej Marchel, Jan Komorowski, Bozena Kaminska, Bartek Wilczyński

**Affiliations:** 1 Nencki Institute of Experimental Biology of the Polish Academy of Sciences, Warsaw, Poland; 2 Faculty of Mathematics, Informatics and Mechanics, University of Warsaw, Warsaw, Poland; 3 Institute of Computer Science of the Polish Academy of Sciences, Warsaw, Poland; 4 Department of Cell and Molecular Biology, Uppsala University, Uppsala, Sweden; 5 Departments of Neurology, Neurosurgery, Neuropathology, The Children’s Memorial Health Institute, Warsaw, Poland; 6 Department of Neurosurgery, Medical University of Warsaw, Warsaw, Poland; 7 Present Address: Institute for Research in Biomedicine (IRB Barcelona), The Barcelona Institute of Science and Technology, Barcelona, Spain; 8 Present Address: Medical University of Gdansk, International Research Agenda 3P Medicine Laboratory, Gdansk, Poland

**Keywords:** Cancer genomics, Epigenetics, Transcriptomics

Correction to: *Nature Communications* 10.1038/s41467-021-23922-2, published online 15 June 2021.

In this article the grant number ETIUDA UMO-2018/28/T/NZ2/00510 relating to Nencki Institute of Experimental Biology for Karolina Stępniak was omitted. The original article has been corrected.

