# Supplementary material for: Mapping chromatin accessibility and active regulatory elements reveals pathological mechanisms in human gliomas
Source: Nat Commun. 2021 Jun 15;12:3621. doi: 10.1038/s41467-021-23922-2 (PMC8206121; doi:10.1038/s41467-021-23922-2)
Supplement: Supplementary file 1 — Supplementary Information [file 41467_2021_23922_MOESM1_ESM.pdf]

A

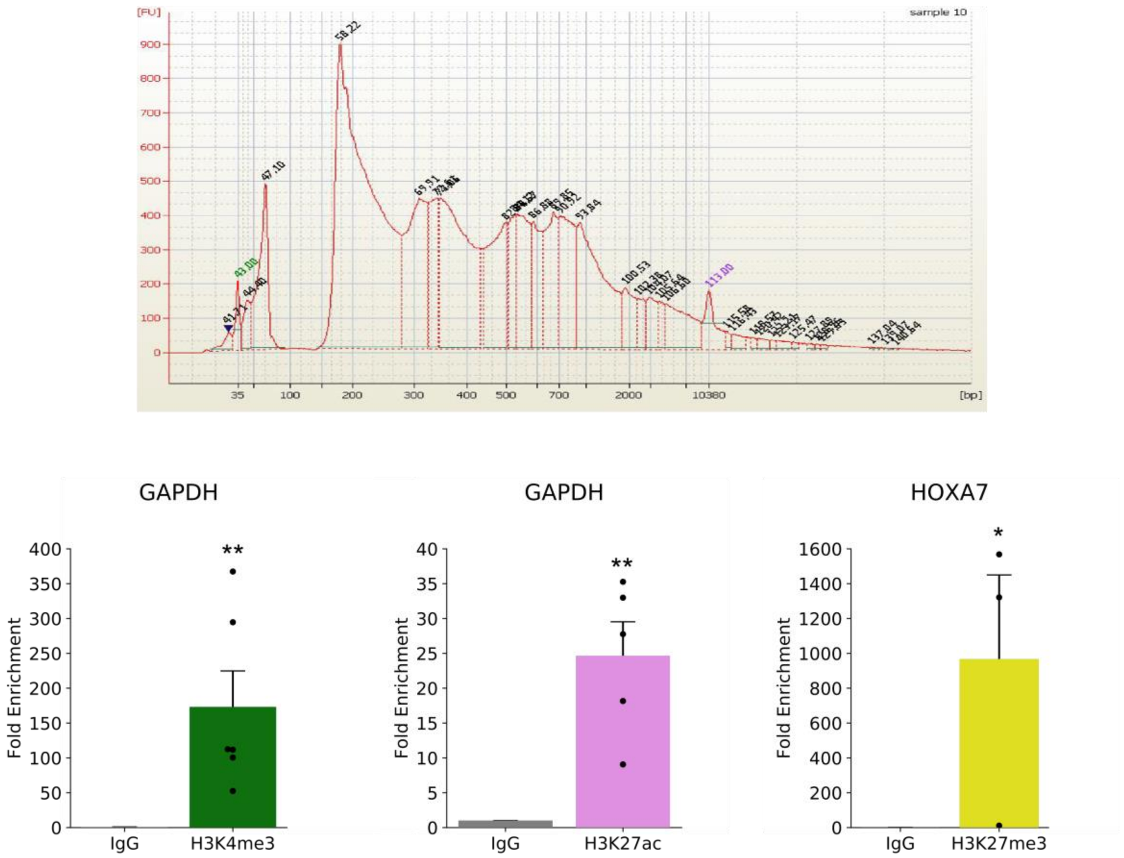

Supplementary Figure 1. Validation experiments results for ATAC-seq and ChIP-seq methods.

A. A characteristic appearance of a single nucleosome and their multiplications in the material isolated for ATAC-seq separated on DNA Agilent chips (PA04 sample).

B. Validation of immunoprecipitation efficiency with antibodies used in the experiments (for details see Methods). Chromatin immunoprecipitation (ChIP)-quantitative polymerase chain reaction (qPCR) analysis of histone modification enrichment in control regions: GAPDH promoter (active chromatin marks, H3K4me3, H3K27ac) and HOXA7 gene body (repressive mark, H3K27me3). Results are calculated as fold enrichment over negative control (immunoprecipitation with normal IgG) and represented as mean ± SEM (H3K4me3 ChIP-qPCR n=6, H3K27ac ChIP-qPCR n=5, H3K27me3 ChIP-qPCR n=3). Statistical significance *P < 0.05 and **P < 0.01 (Mann-Whitney U test, two-tailed). P-values from the paired t-test, two-sided: 0.021 (H3K4me3 ChIP-qPCR), 0.0084 (H3K27ac ChIP-qPCR), 0.18 (H3K27me3 ChIP-qPCR).

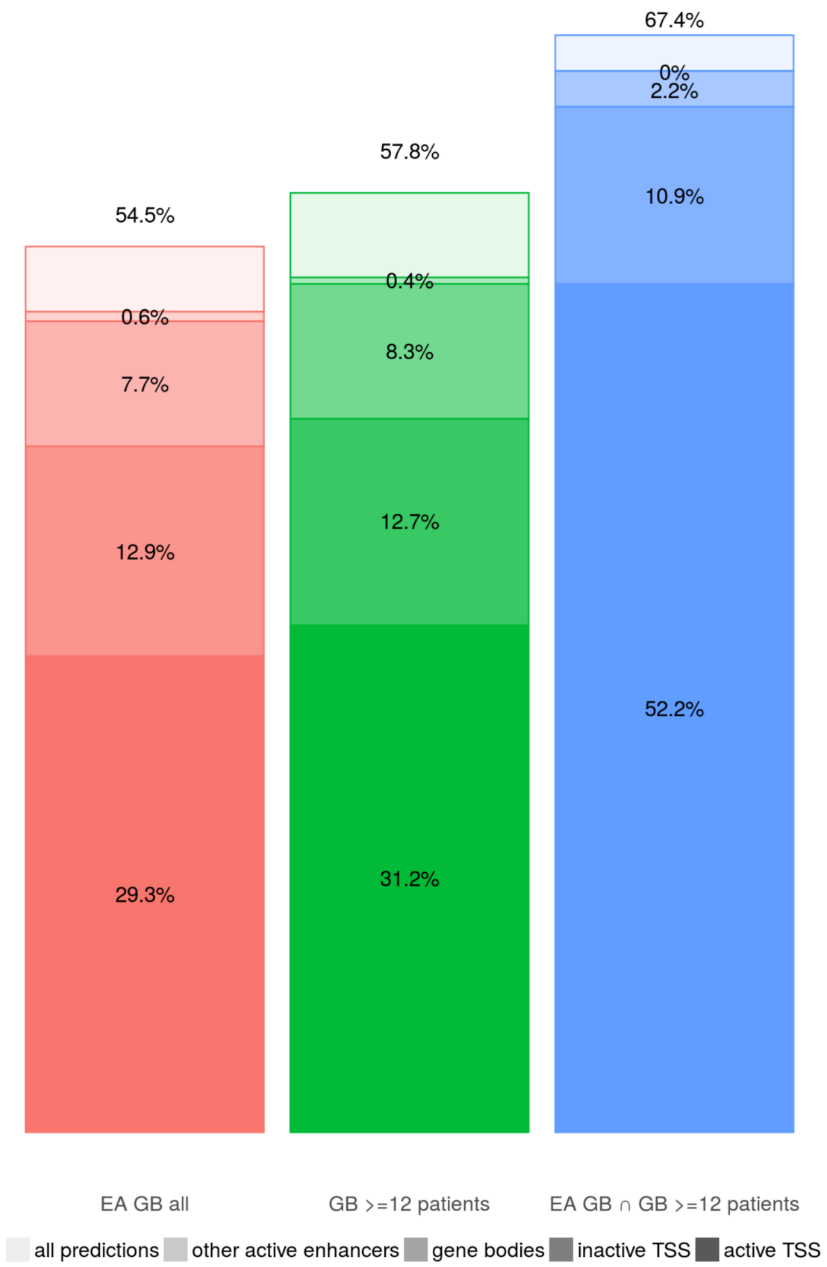

Supplementary Figure 2. Percentage of enhancer bins predicted to be in contact with different genomic elements.

Percentage of occurrence in all enhancer bins for FDR threshold 0.01. Chromatin contacts identified based on developing human brain Hi-C data from Won et al. 2016. *EA GB all* - all glioblastoma enhancers reported in EnhancerAtlas (Gao et al. 2016), the second version; *GB >= 12 patients* - enhancers identified in this work in at least 12 samples; *EA GB and GB >=12* - enhancers present in the same bins in both the glioblastoma set of the second version of EnhancerAtlas (Gao et al. 2016) and at least 12 samples in this work.

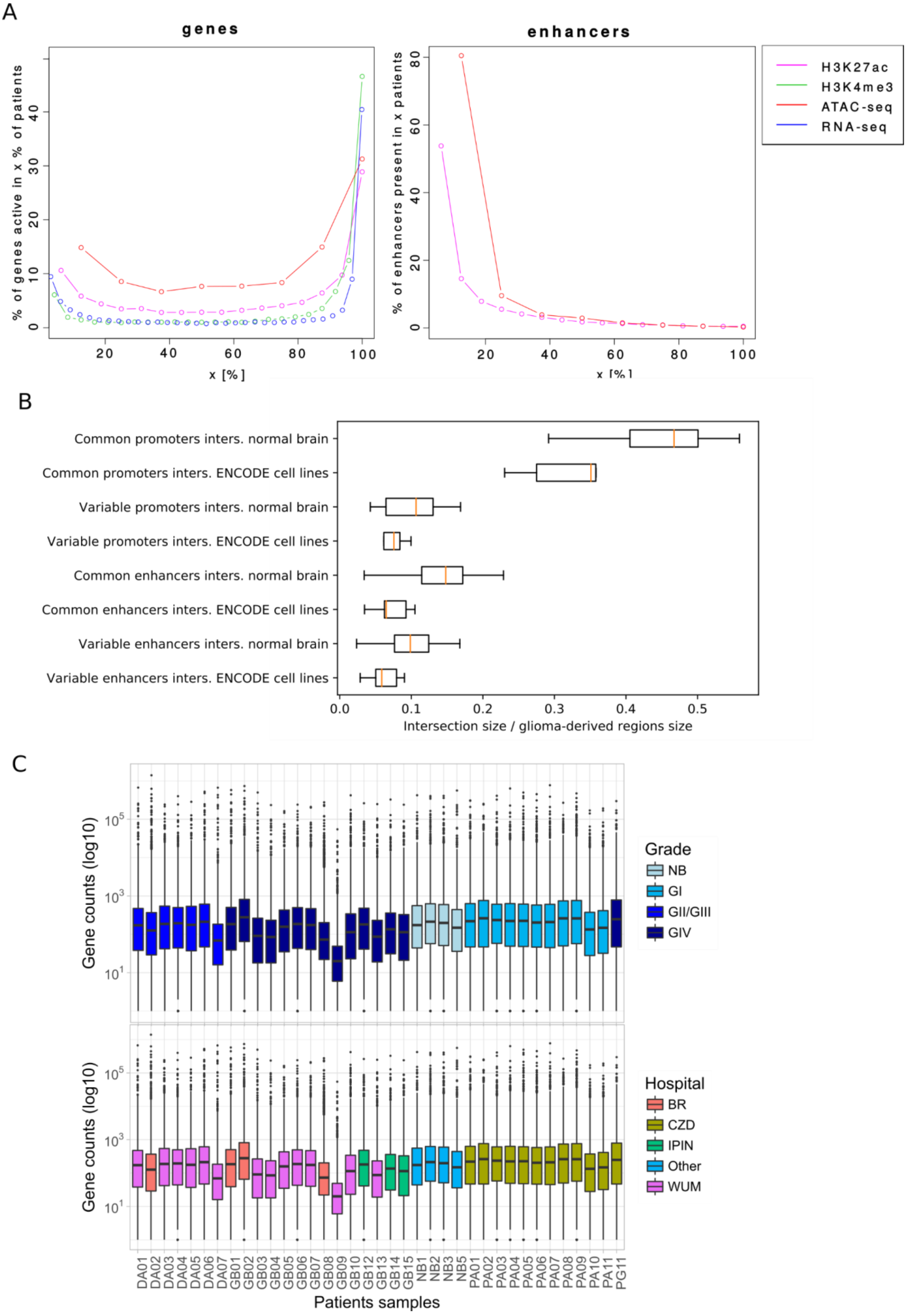
A
genes
% of genes active in x % of patients
x [%]
enhancers
% of enhancers present in x patients
x [%]
H3K27ac
H3K4me3
ATAC-seq
RNA-seq
B
Common promoters inters. normal brain
Common promoters inters. ENCODE cell lines
Variable promoters inters. normal brain
Variable promoters inters. ENCODE cell lines
Common enhancers inters. normal brain
Common enhancers inters. ENCODE cell lines
Variable enhancers inters. normal brain
Variable enhancers inters. ENCODE cell lines
Intersection size / glioma-derived regions size
C
Gene counts (log10)
Grade
NB
GI
GII/GIII
GIV
Hospital
BR
CZD
IPIN
Other
WUM
DA01 DA02 DA03 DA04 DA05 DA06 DA07 GB01 GB02 GB03 GB04 GB05 GB06 GB07 GB08 GB09 GB10 GB12 GB13 GB14 GB15 NB1 NB2 NB3 NB5 PA01 PA02 PA03 PA04 PA05 PA06 PA07 PA08 PA09 PA10 PA11 PG11
Patients samples

Supplementary Figure 3. Global characterization of chromatin structure and its relationship with gene expression.

A. Overlap of active genes and enhancers sets between patients. Colors indicate experimental method used to detect the activity.

B. Size of intersections between H3K27ac ChIP-seq peaks in active promoters and enhancers identified in this work (glioma-derived) and H3K27ac peaks from normal brain (control samples from Sun et al. 2016) or non-brain cell lines from the ENCODE project (Gerstein et al. 2012) vs total size of promoter/enhancer peaks detected in this work.

C. Comparison of RNA-seq results between different glioma Grades and hospitals where brain samples have been collected.

In panels B and C data are represented as boxplots in which the box shows the quartiles of the dataset, the middle line is the median and the whiskers extends to the largest or smallest value no further than 1.5 × the inter-quartile range.

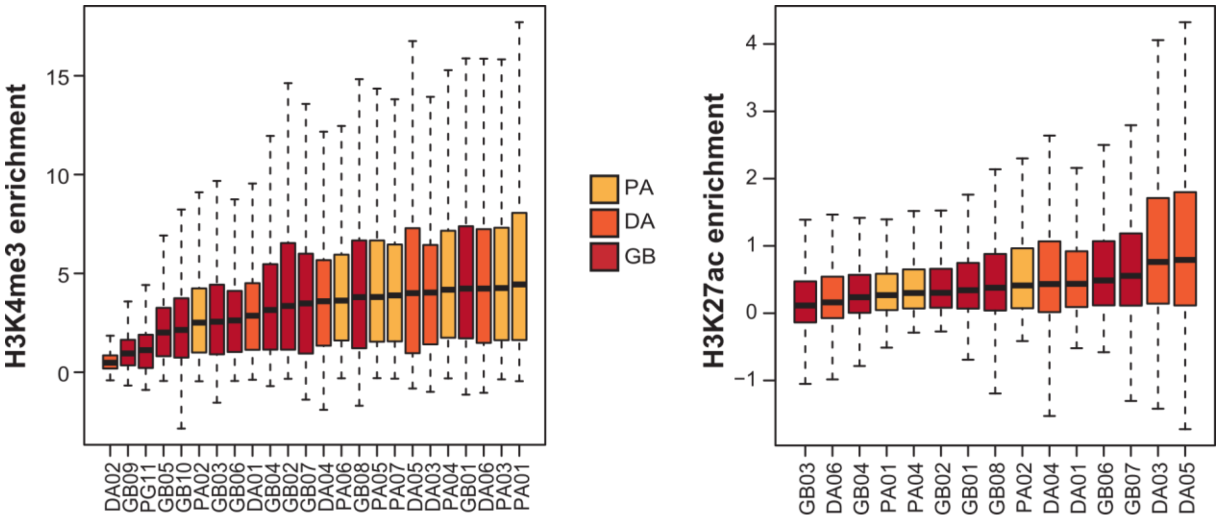

Supplementary Figure 4. Global enrichment in H3K4me3 and H3K27ac in the regions around TSS.

Boxplots representing global enrichment in H3K4me3 (left) and H3K27ac (right) in the regions around TSS in every single glioma sample form our cohort order from lowest to highest median value. The box shows the quartiles of the dataset, the middle line is the median and the whiskers extends to the largest or smallest value no further than 1.5 × the inter-quartile range. The color coding has been used to discriminate between major malignancy groups.

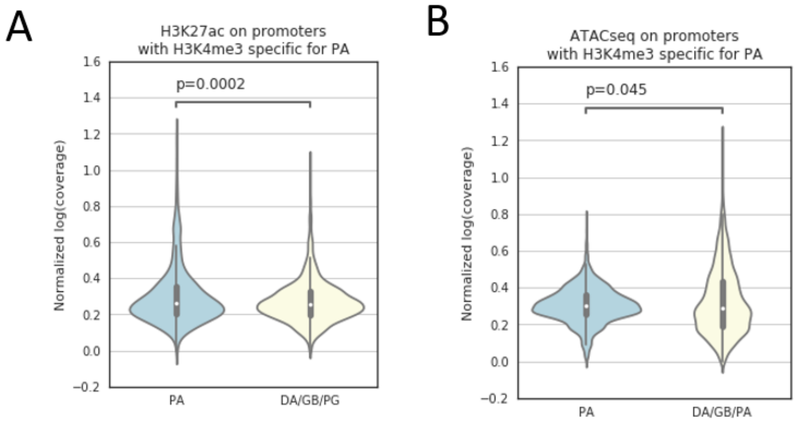

C

Rank	Target	PWM	Motif ID	Raw score	P-value	In top motifs
1	MA0506.1 NRF1		MA0506.1 NRF1	17	6.82e−141	40 %
2	MA1122.1 TFDP1		MA1122.1 TFDP1	3.89	6.32e−134	39 %
3	MA0632.1 Tcfl5		MA0632.1 Tcfl5	1.44	2.24e−129	40 %
4	MA0470.1 E2F4		MA0470.1 E2F4	6.23	4.86e−109	39 %
5	MA0131.2 HINFP		MA0131.2 HINFP	2.51	2.46e−104	33 %
6	MA1099.1 Hes1		MA1099.1 Hes1	1.37	4.43e−102	32 %
7	MA0516.1 SP2		MA0516.1 SP2	95	8.94e−101	31 %
8	MA0162.3 EGR1		MA0162.3 EGR1	1.74	2e−98	28 %
9	MA0732.1 EGR3		MA0732.1 EGR3	4.99	4.81e−93	27 %
10	MA0527.1 ZBTB33		MA0527.1 ZBTB33	1.14	1.88e−80	26 %

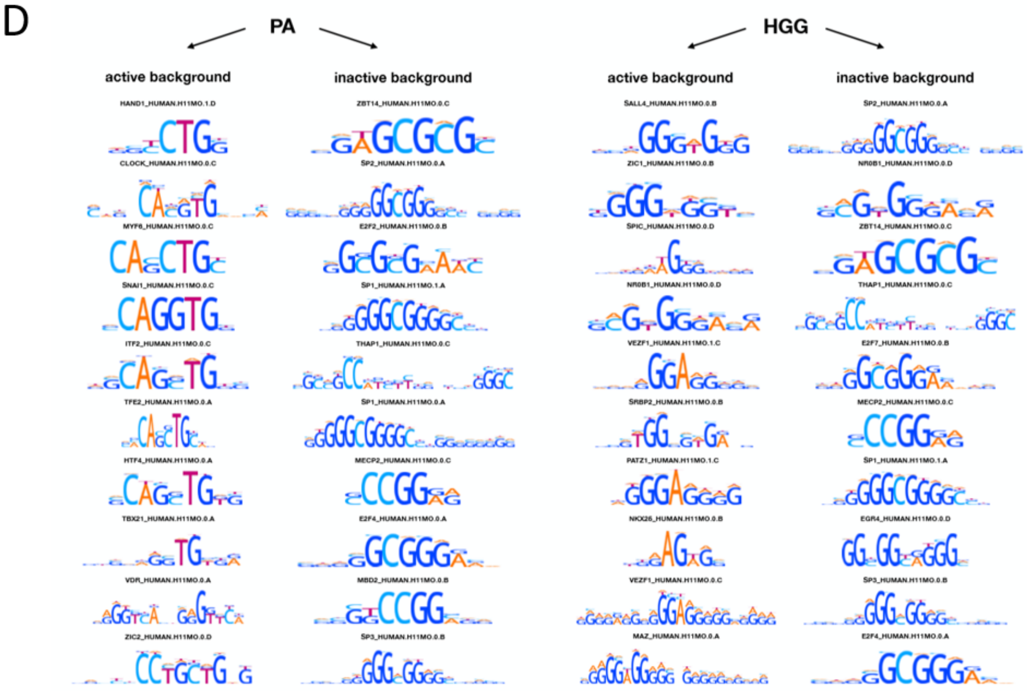

E

	Homo sapiens (REF)	upload_1 (▽ Hierarchy NEW! ?)					
GO biological process complete	#	#	expected	Fold Enrichment	+/-	raw P value	FDR
locomotory behavior	192	8	.85	9.43	+	2.86E-06	4.06E-03
↳behavior	555	12	2.45	4.89	+	7.14E-06	7.43E-03
sodium ion transport	169	7	.75	9.37	+	1.28E-05	1.17E-02
↳monovalent inorganic cation transport	415	10	1.83	5.45	+	1.78E-05	1.54E-02
↳cation transport	819	16	3.62	4.42	+	6.74E-07	1.76E-03
↳ion transport	1284	18	5.67	3.17	+	1.28E-05	1.25E-02
↳metal ion transport	630	16	2.78	5.75	+	2.07E-08	1.08E-04
sensory perception of sound	148	6	.65	9.17	+	6.14E-05	4.36E-02
↳nervous system process	1335	17	5.90	2.88	+	7.59E-05	4.94E-02
↳system process	1893	21	8.37	2.51	+	6.96E-05	4.73E-02
↳multicellular organismal process	6695	58	29.59	1.96	+	1.90E-09	1.48E-05
inorganic cation transmembrane transport	569	13	2.51	5.17	+	1.58E-06	3.09E-03
↳cation transmembrane transport	613	13	2.71	4.80	+	3.52E-06	4.23E-03
↳ion transmembrane transport	886	16	3.92	4.09	+	1.85E-06	3.21E-03
↳transmembrane transport	1208	17	5.34	3.18	+	2.20E-05	1.72E-02
↳inorganic ion transmembrane transport	650	14	2.87	4.87	+	1.20E-06	2.67E-03
epithelium development	1075	16	4.75	3.37	+	2.04E-05	1.67E-02
↳anatomical structure development	5189	46	22.93	2.01	+	3.50E-07	1.09E-03
↳developmental process	5527	52	24.43	2.13	+	1.80E-09	2.81E-05
cell differentiation	3574	35	15.80	2.22	+	2.27E-06	3.54E-03
↳cellular developmental process	3665	35	16.20	2.16	+	3.46E-06	4.50E-03
animal organ development	3077	29	13.60	2.13	+	4.78E-05	3.55E-02
↳system development	4244	38	18.76	2.03	+	6.15E-06	6.86E-03
↳multicellular organism development	4820	46	21.30	2.16	+	3.27E-08	1.28E-04

	Homo sapiens (REF)	upload_1 (▽ Hierarchy NEW! ?)					
GO molecular function complete	#	#	expected	Fold Enrichment	+/-	raw P value	FDR
luteinizing hormone receptor activity	2	2	.01	> 100	+	1.14E-04	3.80E-02
catecholamine binding	19	3	.08	35.72	+	1.20E-04	3.71E-02
solute:sodium symporter activity	68	6	.30	19.96	+	8.98E-07	1.39E-03
↳sodium ion transmembrane transporter activity	147	7	.65	10.77	+	5.33E-06	4.14E-03
↳monovalent inorganic cation transmembrane transporter activity	380	10	1.68	5.95	+	8.45E-06	4.37E-03
↳inorganic cation transmembrane transporter activity	585	14	2.59	5.41	+	3.51E-07	8.16E-04
↳cation transmembrane transporter activity	641	14	2.83	4.94	+	1.02E-06	1.18E-03
↳ion transmembrane transporter activity	896	15	3.96	3.79	+	9.89E-06	4.60E-03
↳transmembrane transporter activity	1066	16	4.71	3.40	+	1.84E-05	7.13E-03
↳transporter activity	1261	18	5.57	3.23	+	1.00E-05	4.24E-03
↳inorganic molecular entity transmembrane transporter activity	839	15	3.71	4.05	+	4.56E-06	4.24E-03
↳metal ion transmembrane transporter activity	432	14	1.91	7.33	+	9.21E-09	4.29E-05
↳solute:cation symporter activity	99	6	.44	13.71	+	6.99E-06	4.65E-03
↳symporter activity	134	6	.59	10.13	+	3.60E-05	1.29E-02
↳secondary active transmembrane transporter activity	219	8	.97	8.27	+	7.28E-06	4.24E-03

	Homo sapiens (REF)	upload_1 (▽ Hierarchy NEW! ?)					
GO cellular component complete	#	#	expected	Fold Enrichment	+/-	raw P value	FDR
synapse part	745	12	3.29	3.64	+	1.19E-04	4.74E-02
plasma membrane region	1095	15	4.84	3.10	+	9.62E-05	4.78E-02
↳plasma membrane part	2836	28	12.53	2.23	+	2.69E-05	5.36E-02
↳plasma membrane	5592	43	24.72	1.74	+	5.03E-05	3.34E-02
↳cell periphery	5712	44	25.25	1.74	+	3.44E-05	3.42E-02

F

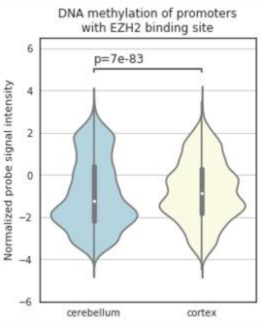

G

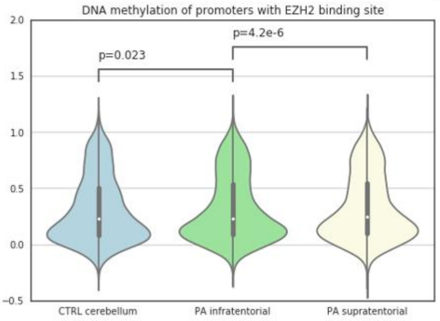

H

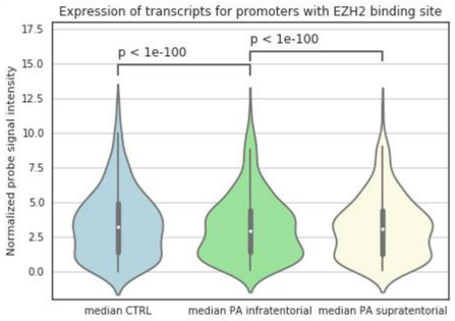

Supplementary Figure 5. Characterization of promoters enriched in H3K4me3 mark in PA samples.

A. H3K27ac ChIP-seq signal on promoters for which H3K4me3 presence is specific for PA compared for PA and higher grade samples.

B. ATAC-seq signal on promoters for which H3K4me3 presence is specific for PA compared for PA and higher grade samples.

C. Transcription factor motifs enrichment in the promoters specifically active in PA compared to promoters inactive in all samples.

D. Transcription factor motifs enrichment in the promoters specifically active in PA or higher Grade gliomas (HGG) compared to promoters inactive in all samples (inactive background) or active in all samples (active background).

E. Enrichment of GO terms calculated for genes specifically active in PA and having the EZH2 protein binding site in promoters.

F. DNA methylation levels on promoters specifically active in PA and having the EZH2 protein binding site. Comparison between control cerebellum and cortex (based on Pidsley et al. 2014)

G. DNA methylation levels on promoters specifically active in PA and having the EZH2 protein binding site. Comparison between control cerebellum and pilocytic astrocytomas located in infra- or supratentorial brain regions (based on Lambert et al. 2013)

H. Expression of transcripts related with promoters specifically active in PA and having the EZH2 protein binding site. Comparison between control cerebellum and pilocytic astrocytomas located in infra- or supratentorial brain regions (based on Lambert et al. 2013).

All p-values were calculated with the one-sided Mann-Whitney U test. In panels A, B, F-H data are represented as violin plots with a nested boxplot, where shape indicates the distribution of data. The box shows the quartiles of the dataset, the middle white dot is the median, whiskers extend to 1.5 × IQR past the low and high quartiles

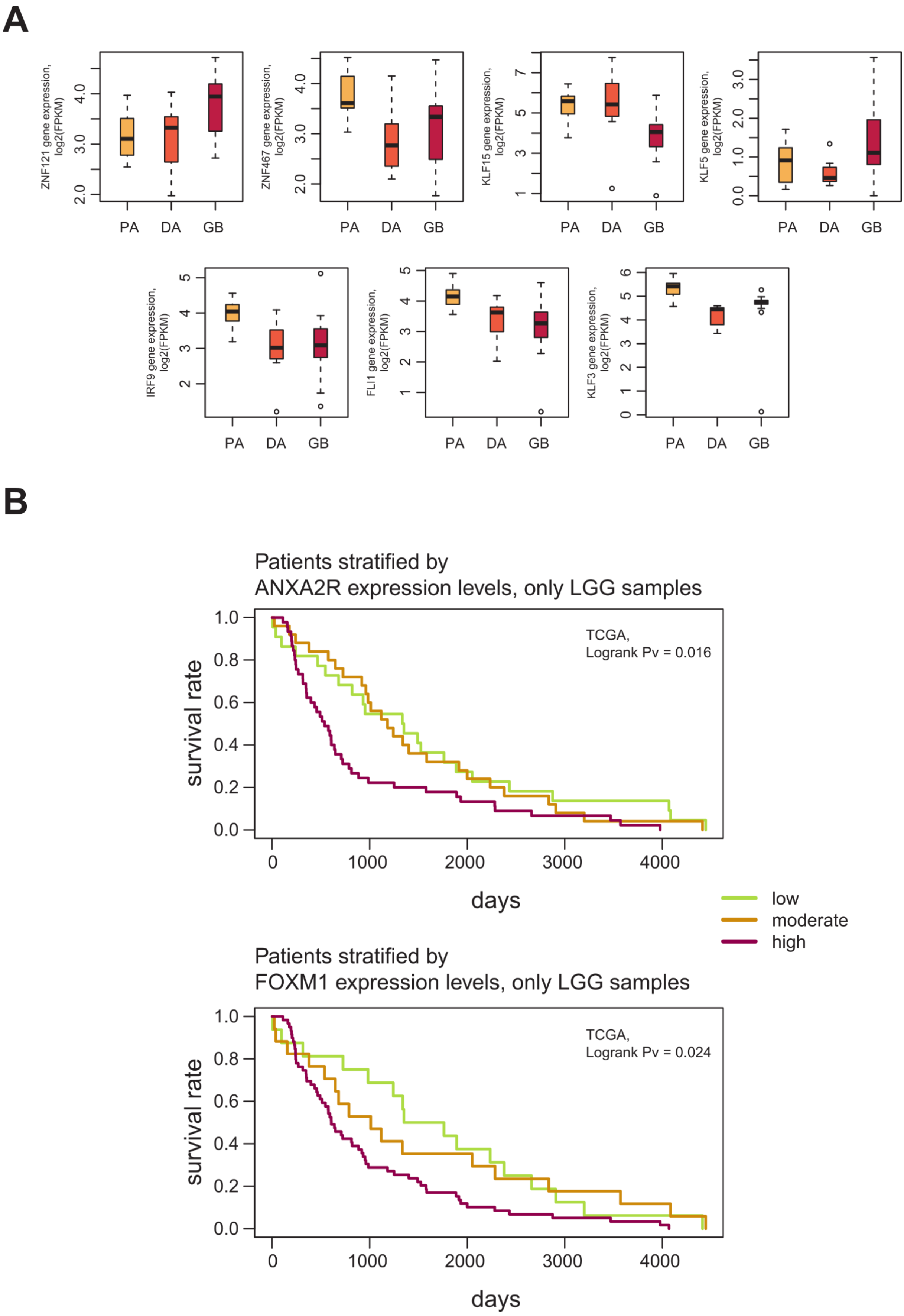
A
ZNF121 gene expression, log2(FPKM)
ZNF467 gene expression, log2(FPKM)
KLF15 gene expression, log2(FPKM)
KLF5 gene expression, log2(FPKM)
IRF9 gene expression, log2(FPKM)
FLI1 gene expression, log2(FPKM)
KLF3 gene expression, log2(FPKM)
PA
DA
GB
B
Patients stratified by ANXA2R expression levels, only LGG samples
TCGA, Logrank Pv = 0.016
survival rate
days
low
moderate
high
Patients stratified by FOXM1 expression levels, only LGG samples
TCGA, Logrank Pv = 0.024
survival rate
days

Supplementary Figure 6. Analysis of putative transcription factors binding in the *ANXA2R* enhancer regions and influence of *ANXA2R* and *FOXM1* expression on patient survival rate.

A. Expression levels of genes coding for transcription factors identified to have a potential binding site in the putative *ANXA2R* enhancer region. Gene expression levels are represented as log-transformed FPKMs. The color coding has been used to discriminate between major malignancy groups. Data are represented as boxplots in which the box shows the quartiles of the dataset, the middle line is the median and the whiskers extends to the largest or smallest value no further than 1.5 × the inter-quartile range.

B. Kaplan-Meier curves plotted for the low grade glioma (LGG) data from TCGA database. The patients were stratified by either by *ANXA2R* (top part) or by *FOXM1* (bottom part) expression levels into three equinumerous sub-groups. Differences in survival rates between sub-groups were assessed with the two-sided log-rank test.

Supplementary Table 1. Clinical data.

Sample	Clinical Diagnosis	Gender	Age	Molecular test results	Tumor Location
PA01	WHO G 1	Juvenile Male	11	IDH wt	infratentorial structure
PA02	WHO G 1	Juvenile Male	6	IDH wt	infratentorial structure
PA03	WHO G 1	Juvenile Female	15	IDH wt	infratentorial structure
PA04	WHO G 1	Juvenile Male	6	IDH wt	infratentorial structure
PA05	WHO G 1	Juvenile Male	17	IDH wt	infratentorial structure
PA06	WHO G 1	Juvenile Female	12	IDH wt	infratentorial structure
PA07	WHO G 1	Juvenile Female	7	IDH wt	infratentorial structure
PA08	WHO G 1	Juvenile Male	5	IDH wt	infratentorial structure
PA09	WHO G 1	Juvenile Female	3	IDH wt	infratentorial structure
PA10	WHO G 1	Juvenile Male	3	IDH wt	infratentorial structure
PA11	WHO G 1	Juvenile Male	15	IDH wt	infratentorial structure
DA01	Astrocytoma anaplasticum WHO G3	Male	27	IDH mut	left frontal lobe
DA02	Difuse Astrocytoma WHO G2/3	Male	41	IDH wt	left frontal lobe
DA03	Astrocytoma anaplasticum WHO G3	Male	49	IDH wt	left frontal lobe
DA04	Astrocytoma WHO G3	Male	66	IDH wt	right frontal lobe
DA05	Oligodenroglioma WHO 2	Male	41	IDH mut	right frontal lobe, right temporal lobe
DA06	Astrocytoma anaplasticum WHO G3	Male	32	IDH mut	right frontal lobe
DA07	Oligoastrocytoma partim anaplasticum WHO G2/3	Male	32	IDH wt	left parietal lobe, right parietal lobe
GB01	Glioblastoma	Male	57	IDH wt	left frontal lobe
GB02	Glioblastoma	Female	62	IDH wt	right parietal lobe
GB03	Glioblastoma	Female	66	IDH wt	right frontal lobe
GB04	Glioblastoma	Male	67	IDH wt	right frontal lobe, right parietal lobe
GB05	Glioblastoma	Male	37	IDH wt	right temporal lobe
GB06	Glioblastoma	Female	53	IDH wt	left temporal lobe
GB07	Glioblastoma	Male	39	IDH mut	left frontal lobe
GB08	Glioblastoma	Female	65	IDH wt	right frontal lobe
GB09	Glioblastoma	Female	65	IDH wt	left frontal lobe
GB10	Glioblastoma	Male	49	IDH wt	right temporal lobe
PG11	WHO G IV, diffuse midline glioma	Juvenile Male	12	IDH wt,	cerebellum
GB12	Glioblastoma	Male	60	IDH wt, H3K27M-mut	non avaiable
GB13	Glioblastoma	Female	66	IDH wt	right temporal lobe
GB14	Glioblastoma	Female	65	IDH wt	non avaiable
GB15	Glioblastoma	Female	45	IDH wt	non avaiable

Supplementary Table 2. Availability of different modality of data for different patient samples.

	patient ID	RNAseq	H3K4me3	H3K27ac	H3K27me3	ATACseq
DA	1	1	1	1	1	1
	2	1	1			1
	3	1	1	1	1	
	4	1	1	1	1	
	5	1	1	1	1	
	6	1	1	1	1	
	7	1				
PA	1	1	1	1		1
	2	1	1	1		1
	3	1	1			1
	4	1	1	1		1
	5	1	1			
	6	1	1			
	7	1	1			
	8	1				
	9	1				
	10	1				
	11	1				
GB	1	1	1	1	1	1
	2	1	1	1		1
	3	1	1	1	1	
	4	1	1	1	1	
	5	1	1	1	1	
	6	1	1	1	1	
	7	1	1	1	1	
	8	1	1	1		
	9	1	1			
	10	1	1			
	12	1				
	13	1				
	14	1				
	15	1				
PG	11	1	1		1	
sum		33	24	16	12	8

Supplementary Table 3. Gene ontology terms enrichment for PA-specific H3K27ac profiles.

GO.name	adj.Pv
trans-synaptic signaling	8.78E-11
chemical synaptic transmission	2.91E-10
anterograde trans-synaptic signaling	2.91E-10
regulation of trans-synaptic signaling	3.33E-06
positive regulation of cell-substrate adhesion	6.41E-06
nervous system development	6.56E-06
regulation of AMPA receptor activity	6.64E-06
modulation of chemical synaptic transmission	6.89E-06

Supplementary Table 4. Promoters specifically marked with H4K3me3 in PA samples.

chr	transcript_start	transcript_end	strand	transcript_id	gene_id	gene_name
chr1	1211326	1214132	-	ENST00000379236	ENSG00000186827	TNFRSF4
chr1	1211340	1214138	-	ENST00000497869	ENSG00000186827	TNFRSF4
chr1	1212019	1213498	-	ENST00000453580	ENSG00000186827	TNFRSF4
chr1	6412418	6424670	-	ENST00000377837	ENSG00000069812	HES2
chr1	6424788	6460944	+	ENST00000377828	ENSG00000187017	ESPN
chr1	10637121	10638763	-	ENST00000478524	ENSG00000130940	CASZ1
chr1	10639241	10654333	+	ENST00000606802	ENSG00000272078	RP4-734G22.3
chr1	12063377	12144207	+	ENST00000263932	ENSG00000120949	TNFRSF8
chr1	12063377	12144205	+	ENST00000417814	ENSG00000120949	TNFRSF8
chr1	12063470	12143117	+	ENST00000514649	ENSG00000120949	TNFRSF8
chr1	16216469	16228027	-	ENST00000602586	ENSG00000237276	ANO7P1
chr1	16754910	16758872	-	ENST00000455405	ENSG00000186715	MST1L
chr1	16758053	16758823	-	ENST00000544155	ENSG00000186715	MST1L
chr1	19265982	19273941	-	ENST00000457194	ENSG00000211454	AKR7L
chr1	19267103	19274194	-	ENST00000429712	ENSG00000211454	AKR7L
chr1	19267130	19274151	-	ENST00000420396	ENSG00000211454	AKR7L
chr1	26037252	26046113	-	ENST00000374278	ENSG00000158014	SLC30A2
chr1	26038022	26046133	-	ENST00000374276	ENSG00000158014	SLC30A2
chr1	26043439	26045451	-	ENST00000498060	ENSG00000158014	SLC30A2
chr1	33307348	33325898	+	ENST00000627421	ENSG00000225313	RP11-415J8.3
chr1	33307366	33325898	+	ENST00000588828	ENSG00000225313	RP11-415J8.3
chr1	33307370	33325955	+	ENST00000624453	ENSG00000225313	RP11-415J8.3
chr1	33307370	33326410	+	ENST00000630229	ENSG00000225313	RP11-415J8.3
chr1	33307371	33326043	+	ENST00000457957	ENSG00000225313	RP11-415J8.3
chr1	34177670	34219131	+	ENST00000488417	ENSG00000142698	C1orf94
chr1	34175866	34175949	-	ENST00000408126	ENSG00000221053	AC115286.1
chr1	34792998	34795747	+	ENST00000342280	ENSG00000187513	GJA4
chr1	34792999	34795090	+	ENST00000450137	ENSG00000187513	GJA4
chr1	40817265	40839289	+	ENST00000443478	ENSG00000117013	KCNQ4
chr1	40818964	40840451	+	ENST00000506017	ENSG00000117013	KCNQ4
chr1	58575423	58577773	-	ENST00000371225	ENSG00000184292	TACSTD2
chr1	62194831	62212328	+	ENST00000498273	ENSG00000240563	L1TD1
chr1	87151298	87151445	+	ENST00000408295	ENSG00000221222	AL139139.1
chr1	108261196	108273689	-	ENST00000438965	ENSG00000238122	RP11-483I13.2
chr1	108272943	108337853	+	ENST00000411846	ENSG00000241361	SLC25A24P1
chr1	108273139	108337387	+	ENST00000434803	ENSG00000241361	SLC25A24P1
chr1	108383736	108421295	-	ENST00000415059	ENSG00000238118	SLC25A24P2
chr1	108420689	108433184	+	ENST00000419814	ENSG00000224698	RP11-131J3.1
chr1	143791542	143791687	-	ENST00000516296	ENSG00000252105	RNU1-143P
chr1	149700151	149700296	-	ENST00000615382	ENSG00000275229	RNU1-68P
chr1	156920650	156933094	+	ENST00000337428	ENSG00000160838	LRRC71
chr1	156920912	156933088	+	ENST00000490146	ENSG00000160838	LRRC71
chr1	179550539	179575933	-	ENST00000367615	ENSG00000116218	NPHS2
chr1	179550539	179575952	-	ENST00000367616	ENSG00000116218	NPHS2
chr1	179576268	179576349	+	ENST00000516066	ENSG00000251875	RNU5F-2P
chr1	179591613	179691270	+	ENST00000367614	ENSG00000162782	TDRD5
chr1	179591622	179691263	+	ENST00000444136	ENSG00000162782	TDRD5
chr1	179591890	179691272	+	ENST00000294848	ENSG00000162782	TDRD5
chr1	182899865	182953023	-	ENST00000483655	ENSG00000157060	SHCBP1L
chr1	182899865	182953370	-	ENST00000367547	ENSG00000157060	SHCBP1L
chr1	182899865	182953525	-	ENST00000488956	ENSG00000157060	SHCBP1L
chr1	182899866	182953350	-	ENST00000423786	ENSG00000157060	SHCBP1L
chr1	182939474	182952749	-	ENST00000467208	ENSG00000157060	SHCBP1L
chr1	182950523	182953111	-	ENST00000497549	ENSG00000157060	SHCBP1L
chr1	183186238	183241261	+	ENST00000493293	ENSG00000058085	LAMC2
chr1	183186288	183244900	+	ENST00000264144	ENSG00000058085	LAMC2
chr1	201283452	201332993	+	ENST00000367324	ENSG00000081277	PKP1

chr1	201283452	201332993	+	ENST00000263946	ENSG00000081277	PKP1
chr1	201283512	201330288	+	ENST00000622031	ENSG00000081277	PKP1
chr1	201283703	201328836	+	ENST00000352845	ENSG00000081277	PKP1
chr1	201380838	201399608	-	ENST00000391967	ENSG00000159166	LAD1
chr1	201381207	201399548	-	ENST00000367313	ENSG00000159166	LAD1
chr1	201386811	201399915	-	ENST00000631576	ENSG00000159166	LAD1
chr1	201386893	201399309	-	ENST00000633953	ENSG00000159166	LAD1
chr1	201399633	201401190	+	ENST00000633182	ENSG00000282221	RP11-27G14.4
chr1	225939432	225940101	-	ENST00000474493	ENSG00000143768	LEFTY2
chr1	226977404	226987544	+	ENST00000485462	ENSG00000163050	ADCK3
chr1	228007051	228061260	+	ENST00000284523	ENSG00000154342	WNT3A
chr1	241749547	241801655	+	ENST00000425826	ENSG00000162843	WDR64
chr2	3702585	3704153	-	ENST00000441632	ENSG00000224661	AC010907.5
chr2	3703592	3847408	+	ENST00000399143	ENSG00000214866	DCDC2C
chr2	3703863	3778867	+	ENST00000423741	ENSG00000214866	DCDC2C
chr2	10090205	10090319	-	ENST00000391256	ENSG00000212558	SNORA26
chr2	21001429	21044073	-	ENST00000233242	ENSG00000084674	APOB
chr2	21001429	21043945	-	ENST00000616098	ENSG00000084674	APOB
chr2	21024170	21043962	-	ENST00000399256	ENSG00000084674	APOB
chr2	25204313	25209202	+	ENST00000431650	ENSG00000230452	LINC01381
chr2	38960504	38973869	+	ENST00000411874	ENSG00000214694	ARHGEF33
chr2	38960505	38973253	+	ENST00000433605	ENSG00000214694	ARHGEF33
chr2	38960561	38969641	+	ENST00000430382	ENSG00000214694	ARHGEF33
chr2	38959287	38960342	-	ENST00000601251	ENSG00000269210	RP11-173C1.1
chr2	48632291	48755724	-	ENST00000602369	ENSG00000279956	RP11-310N16.1
chr2	48686775	48755693	-	ENST00000294954	ENSG00000138039	LHCGR
chr2	48687643	48755671	-	ENST00000405626	ENSG00000138039	LHCGR
chr2	48687669	48755730	-	ENST00000403273	ENSG00000138039	LHCGR
chr2	48687669	48755730	-	ENST00000401907	ENSG00000138039	LHCGR
chr2	48720040	48755569	-	ENST00000428232	ENSG00000138039	LHCGR
chr2	66445193	66567500	+	ENST00000475239	ENSG00000143995	MEIS1
chr2	73254682	73269630	-	ENST00000295133	ENSG00000163013	FBXO41
chr2	74557883	74648037	-	ENST00000290536	ENSG00000159374	M1AP
chr2	74557892	74648338	-	ENST00000536235	ENSG00000159374	M1AP
chr2	74575494	74648338	-	ENST00000438226	ENSG00000159374	M1AP
chr2	74581795	74648309	-	ENST00000422394	ENSG00000159374	M1AP
chr2	74614964	74648330	-	ENST00000421985	ENSG00000159374	M1AP
chr2	86815339	86861924	-	ENST00000393761	ENSG00000172116	CD8B
chr2	86815347	86861915	-	ENST00000393759	ENSG00000172116	CD8B
chr2	86815450	86861915	-	ENST00000349455	ENSG00000172116	CD8B
chr2	86815557	86861915	-	ENST00000331469	ENSG00000172116	CD8B
chr2	86841582	86861924	-	ENST00000390655	ENSG00000172116	CD8B
chr2	86844632	86861924	-	ENST00000431506	ENSG00000172116	CD8B
chr2	86861825	86895042	+	ENST00000616781	ENSG00000233673	ANAPC1P1
chr2	90359809	90360032	-	ENST00000578059	ENSG00000265897	AC018696.1
chr2	106487427	106506940	+	ENST00000417670	ENSG00000254126	CD8BP
chr2	106487429	106504325	+	ENST00000416057	ENSG00000254126	CD8BP
chr2	110083870	110116566	-	ENST00000272462	ENSG00000144063	MALL
chr2	110085023	110116022	-	ENST00000427178	ENSG00000144063	MALL
chr2	110085712	110115821	-	ENST00000424988	ENSG00000144063	MALL
chr2	110732573	111094344	+	ENST00000389811	ENSG00000153093	ACOXL
chr2	110732573	111118222	+	ENST00000439055	ENSG00000153093	ACOXL
chr2	110732634	110888017	+	ENST00000340561	ENSG00000153093	ACOXL
chr2	110732855	110793700	+	ENST00000448863	ENSG00000153093	ACOXL
chr2	127638426	127664419	-	ENST00000476932	ENSG00000072163	LIMS2
chr2	127638426	127664546	-	ENST00000324938	ENSG00000072163	LIMS2
chr2	127638428	127664452	-	ENST00000466410	ENSG00000072163	LIMS2
chr2	130036958	130038422	+	ENST00000447438	ENSG00000231240	KLF2P1
chr2	130212870	130213490	-	ENST00000437982	ENSG00000230756	RHOQP3
chr2	131299220	131300444	-	ENST00000451959	ENSG00000236516	KLF2P4
chr2	131395217	131395542	-	ENST00000413613	ENSG00000226831	MED15P3

chr2	131794961	131801661	-	ENST00000623129	ENSG00000186825	C2orf27B
chr2	161422659	161423135	-	ENST00000444164	ENSG00000224076	AC009487.4
chr2	161422659	161423577	-	ENST00000437683	ENSG00000224076	AC009487.4
chr2	161424015	161428774	+	ENST00000505579	ENSG00000251621	AC009487.5
chr2	161424332	161771035	+	ENST00000482861	ENSG00000144290	SLC4A10
chr2	161424373	161624566	+	ENST00000605990	ENSG00000144290	SLC4A10
chr2	161427453	161651290	+	ENST00000606386	ENSG00000144290	SLC4A10
chr2	185719874	185738721	-	ENST00000421998	ENSG00000226747	AC007966.1
chr2	185736062	185739025	-	ENST00000437717	ENSG00000226747	AC007966.1
chr2	185738628	185833289	+	ENST00000343098	ENSG00000188738	FSIP2
chr2	185738895	185833290	+	ENST00000424728	ENSG00000188738	FSIP2
chr2	185739286	185743294	+	ENST00000465275	ENSG00000188738	FSIP2
chr2	217893473	217978934	-	ENST00000413280	ENSG00000079308	TNS1
chr2	217978707	217992615	+	ENST00000450996	ENSG00000223923	AC010136.2
chr2	218883850	218890099	+	ENST00000483911	ENSG00000135925	WNT10A
chr3	13549131	13638422	+	ENST00000404922	ENSG00000163520	FBLN2
chr3	13549181	13638422	+	ENST00000295760	ENSG00000163520	FBLN2
chr3	13933056	13936944	+	ENST00000611158	ENSG00000275340	FGD5P1
chr3	13934255	13937477	+	ENST00000614866	ENSG00000275340	FGD5P1
chr3	75395188	75395320	+	ENST00000479042	ENSG00000240665	LSP1P2
chr3	75395404	75395536	-	ENST00000487255	ENSG00000242953	RP11-803B1.3
chr3	98657802	98660570	+	ENST00000466609	ENSG00000244153	WWP1P1
chr3	113286930	113441514	-	ENST00000393845	ENSG00000206530	CFAP44
chr3	113362848	113441513	-	ENST00000488854	ENSG00000206530	CFAP44
chr3	113362865	113441610	-	ENST00000295868	ENSG00000206530	CFAP44
chr3	113425982	113441515	-	ENST00000479422	ENSG00000206530	CFAP44
chr3	125958556	125958817	+	ENST00000466506	ENSG00000241439	RP11-666A20.3
chr3	129045763	129046921	+	ENST00000510422	ENSG00000249505	RP11-434H6.2
chr3	129345411	129346164	+	ENST00000603123	ENSG00000270773	RP13-685P2.7
chr3	129974305	129977938	+	ENST00000302649	ENSG00000170893	TRH
chr4	786390	792692	-	ENST00000506404	ENSG00000168993	CPLX1
chr4	904653	915756	-	ENST00000510022	ENSG00000178950	GAK
chr4	904687	915735	-	ENST00000511229	ENSG00000178950	GAK
chr4	911673	915678	-	ENST00000511983	ENSG00000178950	GAK
chr4	911719	914029	-	ENST00000507124	ENSG00000178950	GAK
chr4	8858715	8860827	-	ENST00000557144	ENSG00000258507	RP13-582L3.4
chr4	25655301	25666240	+	ENST00000513204	ENSG00000157765	SLC34A2
chr4	25655822	25676769	+	ENST00000504570	ENSG00000157765	SLC34A2
chr4	25655844	25678748	+	ENST00000382051	ENSG00000157765	SLC34A2
chr4	25656464	25676749	+	ENST00000503434	ENSG00000157765	SLC34A2
chr4	25656469	25666231	+	ENST00000507530	ENSG00000157765	SLC34A2
chr4	48986247	49062079	+	ENST00000226432	ENSG00000109182	CWH43
chr4	48986313	49062081	+	ENST00000514053	ENSG00000109182	CWH43
chr4	48986639	49062076	+	ENST00000513409	ENSG00000109182	CWH43
chr4	76586140	76645354	+	ENST00000490690	ENSG00000138771	SHROOM3
chr4	76586255	76738835	+	ENST00000481002	ENSG00000138771	SHROOM3
chr4	76586286	76645645	+	ENST00000485780	ENSG00000138771	SHROOM3
chr4	76586313	76587265	+	ENST00000484236	ENSG00000138771	SHROOM3
chr4	140442262	140498353	-	ENST00000515354	ENSG00000205301	MGAT4D
chr4	140443198	140498252	-	ENST00000511632	ENSG00000205301	MGAT4D
chr4	140443421	140498370	-	ENST00000515121	ENSG00000205301	MGAT4D
chr4	140443433	140498332	-	ENST00000513106	ENSG00000205301	MGAT4D
chr4	140443436	140498222	-	ENST00000511113	ENSG00000205301	MGAT4D
chr4	140449956	140498222	-	ENST00000503109	ENSG00000205301	MGAT4D
chr4	140464945	140498330	-	ENST00000515402	ENSG00000205301	MGAT4D
chr4	140464981	140498377	-	ENST00000509091	ENSG00000205301	MGAT4D
chr4	140498402	140501330	+	ENST00000624981	ENSG00000280262	RP11-542P2.2
chr4	159228254	159322363	+	ENST00000504604	ENSG00000109756	RAPGEF2
chr4	159229337	159241363	+	ENST00000503328	ENSG00000109756	RAPGEF2
chr5	1392790	1445430	-	ENST00000270349	ENSG00000142319	SLC6A3
chr5	7290823	7296345	+	ENST00000500762	ENSG00000247732	RP11-404K5.1

chr5	73382384	73410509	+	ENST00000505955	ENSG00000249149	RP11-79P5.3
chr5	76170930	76171106	+	ENST00000507884	ENSG00000251668	RP11-466P24.5
chr5	79111779	79132290	+	ENST00000274353	ENSG00000145692	BHMT
chr5	79111809	79116404	+	ENST00000520335	ENSG00000145692	BHMT
chr5	79111809	79119606	+	ENST00000520703	ENSG00000145692	BHMT
chr5	79111809	79131484	+	ENST00000524080	ENSG00000145692	BHMT
chr5	93409359	93570793	-	ENST00000607797	ENSG00000237187	NR2F1-AS1
chr5	93411021	93570819	-	ENST00000606739	ENSG00000237187	NR2F1-AS1
chr5	93411447	93571343	-	ENST00000607831	ENSG00000237187	NR2F1-AS1
chr5	135038831	135040047	-	ENST00000507035	ENSG00000249082	C5orf66-AS1
chr5	135038841	135039696	-	ENST00000511256	ENSG00000249082	C5orf66-AS1
chr5	141387698	141392422	+	ENST00000615384	ENSG00000253953	PCDHGB4
chr5	141387885	141512979	+	ENST00000519479	ENSG00000253953	PCDHGB4
chr5	148321203	148339722	+	ENST00000511717	ENSG00000204909	SPINK9
chr5	150189957	150211072	+	ENST00000230671	ENSG00000011083	SLC6A7
chr5	150190085	150222788	+	ENST00000524041	ENSG00000011083	SLC6A7
chr5	150190251	150195204	+	ENST00000513403	ENSG00000011083	SLC6A7
chr5	176049678	176062021	-	ENST00000512675	ENSG00000248469	RP11-826N14.2
chr5	176060689	176115867	+	ENST00000510151	ENSG00000182230	FAM153B
chr5	176060723	176075219	+	ENST00000514721	ENSG00000182230	FAM153B
chr5	176061122	176090439	+	ENST00000511680	ENSG00000182230	FAM153B
chr5	176061255	176116015	+	ENST00000512862	ENSG00000182230	FAM153B
chr5	177722295	177783398	-	ENST00000510276	ENSG00000170074	FAM153A
chr5	177724159	177782837	-	ENST00000505531	ENSG00000170074	FAM153A
chr5	177747724	177782965	-	ENST00000503567	ENSG00000170074	FAM153A
chr5	177762929	177783364	-	ENST00000503136	ENSG00000170074	FAM153A
chr5	177779556	177782813	-	ENST00000506229	ENSG00000170074	FAM153A
chr5	177782197	177794396	+	ENST00000502515	ENSG00000249109	RP11-1026M7.2
chr6	26611903	26613109	-	ENST00000602994	ENSG00000271071	RP11-457M11.6
chr6	28977475	28977773	+	ENST00000580476	ENSG00000263426	RN7SL471P
chr6	28977613	28977709	+	ENST00000625416	ENSG00000280628	AL662791.1
chr6	33064569	33080710	-	ENST00000419277	ENSG00000231389	HLA-DPA1
chr6	33069019	33080767	-	ENST00000453337	ENSG00000231389	HLA-DPA1
chr6	33069104	33080755	-	ENST00000476642	ENSG00000231389	HLA-DPA1
chr6	33073502	33080775	-	ENST00000417724	ENSG00000231389	HLA-DPA1
chr6	33080386	33084012	+	ENST00000471184	ENSG00000223865	HLA-DPB1
chr6	33080386	33084957	+	ENST00000488575	ENSG00000223865	HLA-DPB1
chr6	33080443	33085231	+	ENST00000498038	ENSG00000223865	HLA-DPB1
chr6	33080487	33085889	+	ENST00000428835	ENSG00000223865	HLA-DPB1
chr6	33080672	33087201	+	ENST00000416804	ENSG00000223865	HLA-DPB1
chr6	33080941	33083890	+	ENST00000478189	ENSG00000223865	HLA-DPB1
chr6	35118071	35141323	-	ENST00000412155	ENSG00000124678	TCP11
chr6	35118071	35141336	-	ENST00000373979	ENSG00000124678	TCP11
chr6	35118072	35141253	-	ENST00000504758	ENSG00000124678	TCP11
chr6	35118072	35141264	-	ENST00000505911	ENSG00000124678	TCP11
chr6	35118072	35141349	-	ENST00000455706	ENSG00000124678	TCP11
chr6	35118072	35141647	-	ENST00000311875	ENSG00000124678	TCP11
chr6	35118074	35141339	-	ENST00000444780	ENSG00000124678	TCP11
chr6	35118077	35141410	-	ENST00000611141	ENSG00000124678	TCP11
chr6	35118077	35141410	-	ENST00000244645	ENSG00000124678	TCP11
chr6	35118135	35141371	-	ENST00000373974	ENSG00000124678	TCP11
chr6	35118269	35141027	-	ENST00000512012	ENSG00000124678	TCP11
chr6	35119240	35141353	-	ENST00000427376	ENSG00000124678	TCP11
chr6	35120186	35141310	-	ENST00000486638	ENSG00000124678	TCP11
chr6	35120429	35141339	-	ENST00000469514	ENSG00000124678	TCP11
chr6	35122199	35141353	-	ENST00000492680	ENSG00000124678	TCP11
chr6	35122252	35141336	-	ENST00000507706	ENSG00000124678	TCP11
chr6	35122286	35141264	-	ENST00000479418	ENSG00000124678	TCP11
chr6	35122297	35141313	-	ENST00000505400	ENSG00000124678	TCP11
chr6	35122305	35141311	-	ENST00000509988	ENSG00000124678	TCP11
chr6	35122309	35141316	-	ENST00000505335	ENSG00000124678	TCP11

chr6	35128358	35141313	-	ENST00000445851	ENSG00000124678	TCP11
chr6	35128689	35141313	-	ENST00000510465	ENSG00000124678	TCP11
chr6	35128705	35141313	-	ENST00000394696	ENSG00000124678	TCP11
chr6	35129115	35141311	-	ENST00000503908	ENSG00000124678	TCP11
chr6	35136127	35141027	-	ENST00000503195	ENSG00000124678	TCP11
chr6	35137746	35141311	-	ENST00000514751	ENSG00000124678	TCP11
chr6	35497874	35512876	-	ENST00000322263	ENSG00000112041	TULP1
chr6	35497874	35512938	-	ENST00000229771	ENSG00000112041	TULP1
chr6	35497877	35512869	-	ENST00000614066	ENSG00000112041	TULP1
chr6	35503751	35511967	-	ENST00000373892	ENSG00000112041	TULP1
chr6	35509724	35512866	-	ENST00000428978	ENSG00000112041	TULP1
chr6	35510706	35512274	-	ENST00000448446	ENSG00000112041	TULP1
chr6	37646303	37648630	-	ENST00000510077	ENSG00000112139	MDGA1
chr6	100388872	100463677	-	ENST00000262901	ENSG00000112246	SIM1
chr6	107634990	107636316	+	ENST00000494935	ENSG00000112320	SOBP
chr6	108176104	108178147	+	ENST00000484978	ENSG00000112333	NR2E1
chr6	133837184	133851800	+	ENST00000412745	ENSG00000223586	LINC01312
chr6	138795926	138904070	+	ENST00000423192	ENSG00000203734	ECT2L
chr6	138796111	138904070	+	ENST00000367682	ENSG00000203734	ECT2L
chr6	138796111	138904070	+	ENST00000541398	ENSG00000203734	ECT2L
chr6	138796120	138838404	+	ENST00000401414	ENSG00000203734	ECT2L
chr7	997607	1055306	-	ENST00000444428	ENSG00000146540	C7orf50
chr7	1055279	1056552	+	ENST00000474396	ENSG00000164849	GPR146
chr7	1055285	1059261	+	ENST00000397095	ENSG00000164849	GPR146
chr7	1056657	1057631	+	ENST00000427680	ENSG00000164849	GPR146
chr7	1057505	1059261	+	ENST00000297468	ENSG00000164849	GPR146
chr7	4857733	4861994	-	ENST00000404991	ENSG00000218823	PAPOLB
chr7	16849957	16850725	-	ENST00000603589	ENSG00000270593	RP11-455J15.1
chr7	39609610	39610290	-	ENST00000420748	ENSG00000231951	AC004837.4
chr7	39609717	39610280	+	ENST00000446267	ENSG00000106540	AC004837.3
chr7	47052793	47053674	+	ENST00000315132	ENSG00000214754	AC004870.5
chr7	56175634	56176412	+	ENST00000450065	ENSG00000233028	RP4-725G10.4
chr7	63789909	63790098	+	ENST00000440057	ENSG00000227397	CTD-2526L21.3
chr7	73312911	73325571	-	ENST00000453152	ENSG00000146755	TRIM50
chr7	73700287	73704259	-	ENST00000480126	ENSG00000106089	STX1A
chr7	73700777	73704055	-	ENST00000497980	ENSG00000106089	STX1A
chr7	73700783	73704496	-	ENST00000491427	ENSG00000106089	STX1A
chr7	73702537	73704191	-	ENST00000461441	ENSG00000106089	STX1A
chr7	76234982	76260034	+	ENST00000479294	ENSG00000177679	SRRM3
chr7	84939335	84940256	-	ENST00000623112	ENSG00000280325	AC074183.3
chr7	84939349	84940245	-	ENST00000439105	ENSG00000232019	AC074183.4
chr7	103352730	103446151	-	ENST00000339444	ENSG00000170615	SLC26A5
chr7	103352730	103446151	-	ENST00000356767	ENSG00000170615	SLC26A5
chr7	103352730	103446151	-	ENST00000393735	ENSG00000170615	SLC26A5
chr7	103374207	103446177	-	ENST00000306312	ENSG00000170615	SLC26A5
chr7	103374212	103446177	-	ENST00000432958	ENSG00000170615	SLC26A5
chr7	103374216	103446177	-	ENST00000354356	ENSG00000170615	SLC26A5
chr7	103388523	103446177	-	ENST00000487407	ENSG00000170615	SLC26A5
chr7	103445207	103514007	+	ENST00000422488	ENSG00000234715	CTB-107G13.1
chr7	131910220	131948953	+	ENST00000415393	ENSG00000224865	AC009518.4
chr7	143391112	143408892	-	ENST00000275815	ENSG00000146904	EPHA1
chr7	143391411	143408805	-	ENST00000488068	ENSG00000146904	EPHA1
chr7	143400826	143407650	-	ENST00000497891	ENSG00000146904	EPHA1
chr7	143407813	143523449	+	ENST00000429289	ENSG00000229153	EPHA1-AS1
chr7	143407833	143415830	+	ENST00000421648	ENSG00000229153	EPHA1-AS1
chr7	143510933	143511911	-	ENST00000439431	ENSG00000176510	OR10AC1
chrX	21654690	21658330	-	ENST00000379499	ENSG00000185915	KLHL34
chrX	24558087	24672677	-	ENST00000379145	ENSG00000102230	PCYT1B
chrX	101191115	101191409	-	ENST00000490943	ENSG00000231643	GHc-210E9.2
chrX	101268860	101291336	-	ENST00000356784	ENSG00000102387	TAF7L
chrX	109623700	109625172	-	ENST00000372101	ENSG00000176076	KCNE5

chrX	126549383	126552851	-	ENST00000371126	ENSG00000198889	DCAF12L1
chrX	153672137	153674911	-	ENST00000418241	ENSG00000130822	PNCK
chr8	1373685	1381394	+	ENST00000633906	ENSG00000282160	AF067845.2
chr8	11708233	11758522	+	ENST00000532059	ENSG00000136574	GATA4
chr8	11708313	11759131	+	ENST00000622443	ENSG00000136574	GATA4
chr8	37963962	37965744	-	ENST00000520341	ENSG00000188778	ADRB3
chr8	37963000	37966666	-	ENST00000614635	ENSG00000188778	ADRB3
chr8	38543652	38552680	-	ENST00000521623	ENSG00000253361	RP11-675F6.3
chr8	39106990	39151448	+	ENST00000523400	ENSG00000197140	ADAM32
chr8	39107529	39160959	+	ENST00000399831	ENSG00000197140	ADAM32
chr8	39107571	39284909	+	ENST00000437682	ENSG00000197140	ADAM32
chr8	39107608	39284909	+	ENST00000519315	ENSG00000197140	ADAM32
chr8	39107649	39284909	+	ENST00000379907	ENSG00000197140	ADAM32
chr8	39107773	39164792	+	ENST00000522506	ENSG00000197140	ADAM32
chr8	39107956	39151406	+	ENST00000521741	ENSG00000197140	ADAM32
chr8	41653220	41797622	-	ENST00000347528	ENSG00000029534	ANK1
chr8	41653220	41797622	-	ENST00000289734	ENSG00000029534	ANK1
chr8	41788882	41797593	-	ENST00000521407	ENSG00000029534	ANK1
chr8	64379149	64379257	+	ENST00000385081	ENSG00000207816	MIR124-2
chr8	101461177	101492499	-	ENST00000520268	ENSG00000254024	KB-1562D12.1
chr8	101492432	101669726	+	ENST00000251808	ENSG00000083307	GRHL2
chr8	101492439	101492789	+	ENST00000521085	ENSG00000083307	GRHL2
chr8	101492442	101544070	+	ENST00000472106	ENSG00000083307	GRHL2
chr8	101492758	101666893	+	ENST00000395927	ENSG00000083307	GRHL2
chr8	142700111	142705127	+	ENST00000292430	ENSG00000160886	LY6K
chr8	142700113	142702722	+	ENST00000518841	ENSG00000160886	LY6K
chr8	142700114	142703454	+	ENST00000519387	ENSG00000160886	LY6K
chr8	142700472	142704164	+	ENST00000522591	ENSG00000160886	LY6K
chr8	142701599	142703460	+	ENST00000519390	ENSG00000160886	LY6K
chr8	143739396	143742135	+	ENST00000534398	ENSG00000203499	FAM83H-AS1
chr9	35971344	35972318	-	ENST00000444752	ENSG00000213866	YBX1P10
chr9	41126430	41128463	-	ENST00000622588	ENSG00000273514	FOXD4L6
chr9	41898472	41899944	-	ENST00000467854	ENSG00000154529	CNTNAP3B
chr9	65282101	65285209	-	ENST00000377420	ENSG00000204779	FOXD4L5
chr9	65736555	65738784	+	ENST00000377413	ENSG00000184659	FOXD4L4
chr9	68302867	68304905	+	ENST00000342833	ENSG00000187559	FOXD4L3
chr9	91159573	91165658	+	ENST00000423719	ENSG00000235641	LINC00484
chr9	91159601	91162705	+	ENST00000609752	ENSG00000235641	LINC00484
chr9	91160356	91162632	+	ENST00000608240	ENSG00000235641	LINC00484
chr9	95480544	95494401	-	ENST00000553256	ENSG00000185920	PTCH1
chr9	96021014	96027960	-	ENST00000583864	ENSG00000225194	LINC00092
chr9	96025716	96027965	-	ENST00000580908	ENSG00000225194	LINC00092
chr9	97076246	97077601	-	ENST00000436355	ENSG00000228376	GAS2L1P2
chr9	97307304	97309493	+	ENST00000472746	ENSG00000197816	CCDC180
chr9	97307321	97343643	+	ENST00000494917	ENSG00000197816	CCDC180
chr9	97307361	97345813	+	ENST00000460482	ENSG00000197816	CCDC180
chr9	97307659	97376933	+	ENST00000529487	ENSG00000197816	CCDC180
chr9	98080840	98088001	-	ENST00000478530	ENSG00000106785	TRIM14
chr9	120855652	120894896	-	ENST00000616568	ENSG00000119403	PHF19
chr9	128250802	128254162	+	ENST00000625457	ENSG00000106976	DNM1
chr9	128250939	128255248	+	ENST00000630850	ENSG00000106976	DNM1
chr9	137138390	137168762	+	ENST00000371561	ENSG00000176884	GRIN1
chr9	137139175	137165422	+	ENST00000350902	ENSG00000176884	GRIN1
chr9	137139410	137165422	+	ENST00000471122	ENSG00000176884	GRIN1
chr9	137139481	137168755	+	ENST00000371550	ENSG00000176884	GRIN1
chr9	137139481	137168755	+	ENST00000371546	ENSG00000176884	GRIN1
chr9	137139481	137168755	+	ENST00000371555	ENSG00000176884	GRIN1
chr9	137139481	137168755	+	ENST00000371553	ENSG00000176884	GRIN1
chr9	137139481	137168755	+	ENST00000371559	ENSG00000176884	GRIN1
chr9	137139481	137168755	+	ENST00000371560	ENSG00000176884	GRIN1
chr11	637293	640706	+	ENST00000176183	ENSG00000069696	DRD4

chr11	17695022	17697471	+	ENST00000524885	ENSG00000254586	RP11-358H18.3
chr11	17695053	17697297	+	ENST00000524479	ENSG00000254586	RP11-358H18.3
chr11	17695267	17696940	-	ENST00000529781	ENSG00000255335	RP11-358H18.2
chr11	64606174	64713911	-	ENST00000377551	ENSG00000110076	NRXN2
chr11	64606177	64713943	-	ENST00000409571	ENSG00000110076	NRXN2
chr11	64668482	64713097	-	ENST00000437746	ENSG00000110076	NRXN2
chr11	64675177	64713943	-	ENST00000466324	ENSG00000110076	NRXN2
chr11	65538819	65546759	-	ENST00000529189	ENSG00000168056	LTBP3
chr11	65542407	65545770	-	ENST00000525443	ENSG00000168056	LTBP3
chr11	65546196	65547565	-	ENST00000527339	ENSG00000168056	LTBP3
chr11	72579281	72584473	-	ENST00000441209	ENSG00000186642	PDE2A
chr11	72579549	72583563	-	ENST00000542223	ENSG00000186642	PDE2A
chr11	72584551	72590419	-	ENST00000475807	ENSG00000186642	PDE2A
chr11	72585426	72590021	-	ENST00000538299	ENSG00000186642	PDE2A
chr11	72584572	72587979	+	ENST00000545254	ENSG00000256633	RP11-169D4.2
chr11	89785945	89787838	+	ENST00000532228	ENSG00000255162	RP11-358N4.3
chr11	90051837	90053736	-	ENST00000530158	ENSG00000254916	RP11-529A4.12
chr11	113387791	113400418	+	ENST00000303941	ENSG00000170209	ANKK1
chr10	13156684	13158136	-	ENST00000447253	ENSG00000203414	BTBD7P1
chr10	23192327	23194252	+	ENST00000376504	ENSG00000168267	PTF1A
chr10	42242721	42276842	+	ENST00000622823	ENSG00000274167	AL031601.5
chr10	46461099	46465881	-	ENST00000612632	ENSG00000204174	NPY4R
chr10	46461099	46465881	-	ENST00000374312	ENSG00000204174	NPY4R
chr10	46911396	47002322	+	ENST00000374342	ENSG00000204179	PTPN20
chr10	46911428	46999950	+	ENST00000417004	ENSG00000204179	PTPN20
chr10	46911428	47002322	+	ENST00000395722	ENSG00000204179	PTPN20
chr10	46911435	47002320	+	ENST00000374346	ENSG00000204179	PTPN20
chr10	46911438	47002322	+	ENST00000395721	ENSG00000204179	PTPN20
chr10	46911438	47002320	+	ENST00000395725	ENSG00000204179	PTPN20
chr10	46911438	47002320	+	ENST00000374218	ENSG00000204179	PTPN20
chr10	46911781	47000789	+	ENST00000395727	ENSG00000204179	PTPN20
chr10	46911781	47000789	+	ENST00000509900	ENSG00000204179	PTPN20
chr10	46911781	47000789	+	ENST00000503851	ENSG00000204179	PTPN20
chr10	46911781	47000789	+	ENST00000506080	ENSG00000204179	PTPN20
chr10	46911781	47000789	+	ENST00000513266	ENSG00000204179	PTPN20
chr10	46911781	47000789	+	ENST00000502705	ENSG00000204179	PTPN20
chr10	46911781	47000789	+	ENST00000505814	ENSG00000204179	PTPN20
chr10	46911781	47000789	+	ENST00000509774	ENSG00000204179	PTPN20
chr10	46911781	47000789	+	ENST00000511769	ENSG00000204179	PTPN20
chr10	46911781	47000789	+	ENST00000513159	ENSG00000204179	PTPN20
chr10	46911781	47000789	+	ENST00000509599	ENSG00000204179	PTPN20
chr10	46911781	47000789	+	ENST00000508715	ENSG00000204179	PTPN20
chr10	46911781	47000789	+	ENST00000510335	ENSG00000204179	PTPN20
chr10	46911781	47000789	+	ENST00000513156	ENSG00000204179	PTPN20
chr10	47918739	47923524	+	ENST00000576178	ENSG00000264717	CH17-360D5.1
chr10	47918739	47923524	+	ENST00000613306	ENSG00000264717	CH17-360D5.1
chr10	58325614	58327030	-	ENST00000562575	ENSG00000261076	RP11-179B15.6
chr10	65054460	65055003	+	ENST00000604859	ENSG00000270754	NEK4P3
chr10	70432315	70441667	-	ENST00000287139	ENSG00000156574	NODAL
chr10	86630825	86631628	-	ENST00000481963	ENSG00000230507	RPL7AP8
chr10	93061264	93068697	+	ENST00000285949	ENSG00000187553	CYP26C1
chr10	117158656	117169059	-	ENST00000419373	ENSG00000234474	MIR3663HG
chr10	117167678	117167774	-	ENST00000583207	ENSG00000266782	MIR3663
chr10	119818686	119826771	+	ENST00000631485	ENSG00000198825	INPP5F
chr10	119818716	119828325	+	ENST00000369080	ENSG00000198825	INPP5F
chr10	119818810	119823819	+	ENST00000631572	ENSG00000198825	INPP5F
chr10	119819447	119826702	+	ENST00000631555	ENSG00000198825	INPP5F
chr10	119819507	119823897	+	ENST00000490818	ENSG00000198825	INPP5F
chr10	119818866	119819133	+	ENST00000634073	ENSG00000282413	RP11-359E7.3
chr10	123713276	123891984	-	ENST00000615851	ENSG00000121898	CPXM2
chr10	123745636	123891814	-	ENST00000241305	ENSG00000121898	CPXM2

chr10	133230274	133231558	+	ENST00000304477	ENSG00000171794	UTF1
chr10	133520406	133539122	+	ENST00000463117	ENSG00000130649	CYP2E1
chr12	7812512	7872852	-	ENST00000340749	ENSG00000173262	SLC2A14
chr12	7812514	7872824	-	ENST00000543909	ENSG00000173262	SLC2A14
chr12	7812519	7872916	-	ENST00000431042	ENSG00000173262	SLC2A14
chr12	7813529	7873251	-	ENST00000542505	ENSG00000173262	SLC2A14
chr12	7813801	7873039	-	ENST00000396589	ENSG00000173262	SLC2A14
chr12	7814020	7872852	-	ENST00000535295	ENSG00000173262	SLC2A14
chr12	7829772	7872874	-	ENST00000546234	ENSG00000173262	SLC2A14
chr12	7829822	7873027	-	ENST00000542782	ENSG00000173262	SLC2A14
chr12	7829856	7872802	-	ENST00000535344	ENSG00000173262	SLC2A14
chr12	7829924	7872883	-	ENST00000537557	ENSG00000173262	SLC2A14
chr12	7829988	7873114	-	ENST00000535266	ENSG00000173262	SLC2A14
chr12	7831623	7872380	-	ENST00000542916	ENSG00000173262	SLC2A14
chr12	7831649	7872861	-	ENST00000536594	ENSG00000173262	SLC2A14
chr12	7831734	7873484	-	ENST00000535383	ENSG00000173262	SLC2A14
chr12	7832766	7872880	-	ENST00000535587	ENSG00000173262	SLC2A14
chr12	7863437	7872852	-	ENST00000539234	ENSG00000173262	SLC2A14
chr12	7870850	7872864	-	ENST00000544749	ENSG00000173262	SLC2A14
chr12	50841498	50841785	-	ENST00000497925	ENSG00000243075	RN7SL519P
chr12	50842920	50887884	+	ENST00000551456	ENSG00000186452	TMPRSS12
chr12	50842933	50887884	+	ENST00000398458	ENSG00000186452	TMPRSS12
chr12	52250466	52258553	-	ENST00000534226	ENSG00000135477	KRT87P
chr12	52250544	52258867	-	ENST00000529785	ENSG00000135477	KRT87P
chr12	53195654	53197111	-	ENST00000551887	ENSG00000139626	ITGB7
chr12	53196101	53197709	-	ENST00000549196	ENSG00000139626	ITGB7
chr12	98735518	98745839	-	ENST00000551212	ENSG00000185046	ANKS1B
chr12	101155493	101210407	-	ENST00000536262	ENSG00000256870	SLC5A8
chr12	107580650	107659642	+	ENST00000357167	ENSG00000151136	BTBD11
chr12	124529722	124530228	-	ENST00000623659	ENSG00000279527	RP11-83B20.2
chr12	126191378	126225314	+	ENST00000536505	ENSG00000255778	RP4-765H13.1
chr12	130070325	130072685	+	ENST00000535487	ENSG00000256906	RP11-474D1.2
chr13	21319156	21320866	+	ENST00000400595	ENSG00000215571	GRK6P1
chr13	30906191	30925572	+	ENST00000380482	ENSG00000102802	MEDAG
chr13	30906706	30924449	+	ENST00000428944	ENSG00000102802	MEDAG
chr13	53199981	53202753	+	ENST00000428983	ENSG00000225510	PCDH8P1
chr13	53201507	53201984	+	ENST00000623224	ENSG00000225510	PCDH8P1
chr13	94699694	94702862	-	ENST00000433569	ENSG00000238230	LINC00391
chr13	110809676	110813084	-	ENST00000569854	ENSG00000259831	LINC00567
chr13	113130416	113132054	+	ENST00000415696	ENSG00000230371	KARSP2
chr14	21320151	21325867	+	ENST00000553500	ENSG00000092200	RPGRIP1
chr14	21321359	21351301	+	ENST00000555322	ENSG00000092200	RPGRIP1
chr14	21321364	21351301	+	ENST00000555587	ENSG00000092200	RPGRIP1
chr14	21321373	21324898	+	ENST00000554303	ENSG00000092200	RPGRIP1
chr14	37622065	37842463	+	ENST00000553443	ENSG00000139865	TTC6
chr14	64600243	64601644	-	ENST00000556933	ENSG00000259010	RP11-973N13.2
chr14	74239472	74262738	+	ENST00000261980	ENSG00000119614	VSX2
chr14	77265483	77271312	-	ENST00000298352	ENSG00000165553	NGB
chr14	90452063	90455117	-	ENST00000555413	ENSG00000258678	RP11-1078H9.1
chr14	90455230	90458866	+	ENST00000442515	ENSG00000233208	LINC00642
chr14	90455271	90458904	+	ENST00000419459	ENSG00000233208	LINC00642
chr14	90455329	90458905	+	ENST00000444942	ENSG00000233208	LINC00642
chr14	93934167	93940495	-	ENST00000553883	ENSG00000100628	ASB2
chr14	93939555	93944129	+	ENST00000557646	ENSG00000258987	RP11-131H24.4
chr14	96592733	96595762	+	ENST00000553378	ENSG00000258702	RP11-433J8.1
chr14	96592768	96594760	+	ENST00000555496	ENSG00000258702	RP11-433J8.1
chr14	100538939	100540409	+	ENST00000553301	ENSG00000259031	CTD-2062F14.3
chr14	101552221	101560403	-	ENST00000557109	ENSG00000258498	DIO3OS
chr14	101557302	101560348	-	ENST00000617327	ENSG00000258498	DIO3OS
chr14	101557304	101560375	-	ENST00000554441	ENSG00000258498	DIO3OS
chr14	101557304	101560411	-	ENST00000554735	ENSG00000258498	DIO3OS

chr14	101557304	101560422	-	ENST00000555174	ENSG00000258498	DIO3OS
chr14	101557304	101560431	-	ENST00000557661	ENSG00000258498	DIO3OS
chr14	101557308	101560403	-	ENST00000557532	ENSG00000258498	DIO3OS
chr14	101557308	101560403	-	ENST00000554694	ENSG00000258498	DIO3OS
chr14	101558553	101560306	-	ENST00000555882	ENSG00000258498	DIO3OS
chr14	101559588	101560392	-	ENST00000553729	ENSG00000258498	DIO3OS
chr14	101560287	101560422	-	ENST00000408206	ENSG00000277601	MIR1247
chr14	101561351	101563452	+	ENST00000510508	ENSG00000197406	DIO3
chr14	102922656	102931192	+	ENST00000299155	ENSG00000166126	AMN
chr14	102922922	102930841	+	ENST00000541086	ENSG00000166126	AMN
chr14	104085679	104115581	+	ENST00000551177	ENSG00000166183	ASPG
chr14	104085686	104107235	+	ENST00000548372	ENSG00000166183	ASPG
chr14	104085691	104112633	+	ENST00000546892	ENSG00000166183	ASPG
chr14	104117405	104117514	+	ENST00000384836	ENSG00000207568	MIR203A
chr15	45430529	45433342	+	ENST00000396650	ENSG00000166920	C15orf48
chr15	45430579	45448761	+	ENST00000558435	ENSG00000166920	C15orf48
chr15	45430607	45433449	+	ENST00000344300	ENSG00000166920	C15orf48
chr15	45430611	45431325	+	ENST00000558632	ENSG00000166920	C15orf48
chr15	52109263	52112775	-	ENST00000260442	ENSG00000137875	BCL2L10
chr15	52109750	52112768	-	ENST00000561198	ENSG00000137875	BCL2L10
chr15	68930500	68946811	+	ENST00000310673	ENSG00000258484	SPESP1
chr15	68930504	69028889	+	ENST00000557966	ENSG00000137808	RP11-809H16.2
chr15	68930525	69056733	+	ENST00000448182	ENSG00000255346	NOX5
chr15	68930525	69062743	+	ENST00000260364	ENSG00000255346	NOX5
chr15	68930634	69056725	+	ENST00000455873	ENSG00000255346	NOX5
chr15	70570958	70586606	-	ENST00000630447	ENSG00000280639	RP11-96C21.2
chr15	90293939	90294207	+	ENST00000613111	ENSG00000275803	RN7SL736P
chr15	97665624	97874550	-	ENST00000614972	ENSG00000251209	LINC00923
chr15	97742218	97874550	-	ENST00000503768	ENSG00000251209	LINC00923
chr15	97742622	97874550	-	ENST00000503874	ENSG00000251209	LINC00923
chr15	97876289	97878386	+	ENST00000562480	ENSG00000259870	RP11-753A21.2
chr15	100372939	100408787	+	ENST00000560718	ENSG00000259430	CERS3-AS1
chr16	2235689	2236913	-	ENST00000564055	ENSG00000259780	RP11-304L19.12
chr16	2235816	2238711	+	ENST00000564065	ENSG00000167968	DNASE1L2
chr16	2236467	2238711	+	ENST00000320700	ENSG00000167968	DNASE1L2
chr16	2236467	2238708	+	ENST00000613572	ENSG00000167968	DNASE1L2
chr16	2236469	2238711	+	ENST00000567494	ENSG00000167968	DNASE1L2
chr16	2236725	2238711	+	ENST00000382437	ENSG00000167968	DNASE1L2
chr16	2236826	2238000	+	ENST00000569184	ENSG00000167968	DNASE1L2
chr16	2783953	2786841	-	ENST00000576886	ENSG00000103355	PRSS33
chr16	23690638	23694760	-	ENST00000562458	ENSG00000134398	ERN2
chr16	23753689	23755939	-	ENST00000623305	ENSG00000279756	CTD-2385L22.2
chr16	23754627	23758951	+	ENST00000300113	ENSG00000166869	CHP2
chr16	32084274	32084502	-	ENST00000563973	ENSG00000261727	RP11-1166P10.6
chr16	49466106	49469875	+	ENST00000561523	ENSG00000261623	RP11-189E14.4
chr16	53364982	53373083	-	ENST00000565598	ENSG00000260078	RP11-44F14.1
chr16	53373493	53384259	+	ENST00000566383	ENSG00000261804	RP11-44F14.2
chr16	56676132	56677763	+	ENST00000379816	ENSG00000275691	RP11-249C24.12
chr16	63314264	63618046	-	ENST00000568932	ENSG00000260658	RP11-368L12.1
chr16	63531088	63618046	-	ENST00000564290	ENSG00000260658	RP11-368L12.1
chr16	63531233	63618044	-	ENST00000563855	ENSG00000260658	RP11-368L12.1
chr16	66579448	66588275	+	ENST00000379486	ENSG00000140932	CMTM2
chr16	66579457	66588275	+	ENST00000268595	ENSG00000140932	CMTM2
chr16	66579460	66587985	+	ENST00000569316	ENSG00000140932	CMTM2
chr16	69798865	69940178	+	ENST00000544162	ENSG00000198373	WWP2
chr16	69799046	69871865	+	ENST00000567986	ENSG00000198373	WWP2
chr16	81988855	82011488	-	ENST00000328945	ENSG00000184860	SDR42E1
chr16	81999699	82011481	-	ENST00000532128	ENSG00000184860	SDR42E1
chr16	81999755	82011465	-	ENST00000534209	ENSG00000184860	SDR42E1
chr16	89195761	89200121	-	ENST00000614943	ENSG00000259803	SLC22A31
chr16	89199105	89200121	-	ENST00000568161	ENSG00000259803	SLC22A31

chr16	89707784	89710642	-	ENST00000565023	ENSG00000075399	VPS9D1
chr16	89710933	89713080	-	ENST00000568691	ENSG00000075399	VPS9D1
chr16	89710965	89711644	-	ENST00000565452	ENSG00000075399	VPS9D1
chr16	89711359	89713040	-	ENST00000567379	ENSG00000075399	VPS9D1
chr16	89711856	89718165	+	ENST00000562866	ENSG00000261373	VPS9D1-AS1
chr17	1241939	1254000	-	ENST00000575911	ENSG00000262213	AC144836.1
chr17	2720801	2723947	-	ENST00000614400	ENSG00000277200	RP11-74E22.8
chr17	3751870	3754696	-	ENST00000572121	ENSG00000083457	ITGAE
chr17	6684713	6713567	-	ENST00000433363	ENSG00000141485	SLC13A5
chr17	6684723	6713369	-	ENST00000293800	ENSG00000141485	SLC13A5
chr17	6686080	6713366	-	ENST00000381074	ENSG00000141485	SLC13A5
chr17	6686097	6713377	-	ENST00000573648	ENSG00000141485	SLC13A5
chr17	6695817	6713359	-	ENST00000572094	ENSG00000141485	SLC13A5
chr17	6703073	6713369	-	ENST00000572352	ENSG00000141485	SLC13A5
chr17	6703881	6713371	-	ENST00000576323	ENSG00000141485	SLC13A5
chr17	6704036	6713369	-	ENST00000575230	ENSG00000141485	SLC13A5
chr17	6994642	6995189	-	ENST00000573222	ENSG00000262089	RP11-589P10.5
chr17	6996065	7010736	+	ENST00000251535	ENSG00000108839	ALOX12
chr17	6996982	7000469	+	ENST00000480801	ENSG00000108839	ALOX12
chr17	15740628	15749192	-	ENST00000580194	ENSG00000233002	AC005324.6
chr17	15748703	15749315	-	ENST00000587972	ENSG00000267227	RP11-640I15.2
chr17	35459481	35460550	-	ENST00000588022	ENSG00000267312	RP11-1094M14.7
chr17	41523617	41528308	-	ENST00000361566	ENSG00000171345	KRT19
chr17	41524519	41528299	-	ENST00000455635	ENSG00000171345	KRT19
chr17	42038232	42038734	-	ENST00000602842	ENSG00000270010	CTD-2132N18.4
chr17	42549214	42555213	+	ENST00000585807	ENSG00000108786	HSD17B1
chr17	42667914	42669698	-	ENST00000591476	ENSG00000068137	PLEKHH3
chr17	43914433	43917399	-	ENST00000591374	ENSG00000282199	RP11-527L4.5
chr17	43917194	43917985	+	ENST00000632077	ENSG00000267496	FAM215A
chr17	43917209	43917963	+	ENST00000588043	ENSG00000267496	FAM215A
chr17	48133440	48430190	-	ENST00000336915	ENSG00000141293	SKAP1
chr17	48133445	48430275	-	ENST00000584709	ENSG00000141293	SKAP1
chr17	48136938	48430219	-	ENST00000584924	ENSG00000141293	SKAP1
chr17	50111336	50117799	-	ENST00000508892	ENSG00000167100	SAMD14
chr17	50114228	50116287	-	ENST00000515816	ENSG00000167100	SAMD14
chr17	50470931	50473790	+	ENST00000512537	ENSG00000167107	ACSF2
chr17	50834650	50841308	+	ENST00000426127	ENSG00000173714	WFIKKN2
chr17	50835410	50842348	+	ENST00000311378	ENSG00000173714	WFIKKN2
chr17	58275571	58278119	-	ENST00000578493	ENSG00000005381	MPO
chr17	58278837	58279399	-	ENST00000581022	ENSG00000005381	MPO
chr17	58279876	58280687	-	ENST00000580005	ENSG00000005381	MPO
chr17	58420167	58488384	-	ENST00000323777	ENSG00000176160	HSF5
chr17	58420775	58488340	-	ENST00000617946	ENSG00000176160	HSF5
chr17	63849292	63862531	+	ENST00000463377	ENSG00000240280	TCAM1P
chr17	63849313	63859554	+	ENST00000469014	ENSG00000240280	TCAM1P
chr17	66835117	66885486	+	ENST00000533854	ENSG00000075429	CACNG5
chr17	74274247	74314884	+	ENST00000582036	ENSG00000171595	DNAI2
chr17	74274256	74314884	+	ENST00000579055	ENSG00000171595	DNAI2
chr17	74274290	74314884	+	ENST00000311014	ENSG00000171595	DNAI2
chr18	598391	624907	+	ENST00000584370	ENSG00000079101	CLUL1
chr18	3411608	3457684	+	ENST00000552383	ENSG00000177426	TGIF1
chr18	3411927	3458406	+	ENST00000401449	ENSG00000177426	TGIF1
chr18	3412204	3418846	+	ENST00000547233	ENSG00000177426	TGIF1
chr18	3412854	3457574	+	ENST00000550958	ENSG00000177426	TGIF1
chr18	3412856	3458406	+	ENST00000548489	ENSG00000177426	TGIF1
chr18	4775054	5004524	-	ENST00000585028	ENSG00000266268	RP11-172F10.1
chr18	4807935	5004537	-	ENST00000584204	ENSG00000266268	RP11-172F10.1
chr18	5002759	5003254	-	ENST00000577627	ENSG00000264775	PPIAP14
chr18	8360820	8367034	-	ENST00000580491	ENSG00000266149	RP11-789C17.3
chr18	10726632	10728539	+	ENST00000581724	ENSG00000264714	RP11-21G15.1
chr18	13641758	13645712	+	ENST00000592991	ENSG00000168675	LDLRAD4

chr18	30990008	31042741	-	ENST00000360428	ENSG00000134762	DSC3
chr18	30990086	31042815	-	ENST00000434452	ENSG00000134762	DSC3
chr18	57427133	57432112	+	ENST00000620778	ENSG00000277837	RP11-714M23.2
chr18	59969438	59971396	+	ENST00000588207	ENSG00000267497	NFE2L3P1
chr18	79787121	79791432	+	ENST00000587174	ENSG00000266924	RP11-154H12.2
chr20	841275	846279	+	ENST00000541082	ENSG00000125898	FAM110A
chr20	958452	1002261	-	ENST00000217260	ENSG00000101282	RSPO4
chr20	960254	1002264	-	ENST00000400634	ENSG00000101282	RSPO4
chr20	3667965	3673747	-	ENST00000466620	ENSG00000149451	ADAM33
chr20	23035386	23036812	+	ENST00000255008	ENSG00000132671	SSTR4
chr20	58459101	58514938	-	ENST00000371149	ENSG00000198768	APCDD1L
chr20	58467640	58515131	-	ENST00000425773	ENSG00000198768	APCDD1L
chr20	58515379	58619888	+	ENST00000427140	ENSG00000231290	APCDD1L-AS1
chr20	58515499	58523802	+	ENST00000445984	ENSG00000231290	APCDD1L-AS1
chr20	58515499	58524728	+	ENST00000448374	ENSG00000231290	APCDD1L-AS1
chr20	58515499	58547822	+	ENST00000420279	ENSG00000231290	APCDD1L-AS1
chr20	58515543	58526032	+	ENST00000447767	ENSG00000231290	APCDD1L-AS1
chr20	58515568	58522492	+	ENST00000427794	ENSG00000231290	APCDD1L-AS1
chr20	58515727	58573970	+	ENST00000439558	ENSG00000231290	APCDD1L-AS1
chr20	62463497	62475970	-	ENST00000252997	ENSG00000130700	GATA5
chr19	7787549	7790621	+	ENST00000596555	ENSG00000268297	CLEC4GP1
chr19	12465609	12513833	-	ENST00000455490	ENSG00000242852	ZNF709
chr19	12494818	12513854	-	ENST00000493776	ENSG00000242852	ZNF709
chr19	12583485	12640098	-	ENST00000465656	ENSG00000188033	ZNF490
chr19	12643831	12648397	-	ENST00000597692	ENSG00000269242	CTD-2192J16.22
chr19	12646511	12647584	-	ENST00000469423	ENSG00000104774	MAN2B1
chr19	12646513	12647146	-	ENST00000480851	ENSG00000104774	MAN2B1
chr19	12646513	12647673	-	ENST00000493218	ENSG00000104774	MAN2B1
chr19	13933209	13936029	-	ENST00000588764	ENSG00000132000	PODNL1
chr19	14583084	14611157	+	ENST00000547437	ENSG00000187912	CLEC17A
chr19	14583123	14610420	+	ENST00000417570	ENSG00000187912	CLEC17A
chr19	14583123	14610420	+	ENST00000339847	ENSG00000187912	CLEC17A
chr19	14583123	14610420	+	ENST00000551730	ENSG00000187912	CLEC17A
chr19	14584095	14584395	+	ENST00000462468	ENSG00000243488	RN7SL337P
chr19	15508493	15552317	+	ENST00000269703	ENSG00000171954	CYP4F22
chr19	21444241	21463908	-	ENST00000596705	ENSG00000268119	CTD-2561J22.5
chr19	21449129	21463880	-	ENST00000597444	ENSG00000268119	CTD-2561J22.5
chr19	21452040	21463884	-	ENST00000594557	ENSG00000268119	CTD-2561J22.5
chr19	21453026	21463873	-	ENST00000600469	ENSG00000268119	CTD-2561J22.5
chr19	21453196	21463872	-	ENST00000599993	ENSG00000268119	CTD-2561J22.5
chr19	21455331	21463897	-	ENST00000593653	ENSG00000268119	CTD-2561J22.5
chr19	28435388	28727680	-	ENST00000592347	ENSG00000267243	AC005307.3
chr19	28724276	28727777	-	ENST00000586528	ENSG00000267243	AC005307.3
chr19	37007857	37127442	+	ENST00000590332	ENSG00000197050	ZNF420
chr19	37007859	37127255	+	ENST00000587029	ENSG00000197050	ZNF420
chr19	37823722	37855196	-	ENST00000592640	ENSG00000225868	AC016582.2
chr19	37833368	37855215	-	ENST00000433142	ENSG00000225868	AC016582.2
chr19	39246314	39248856	-	ENST00000634967	ENSG00000272395	IFNL4
chr19	39246314	39248856	-	ENST00000634680	ENSG00000272395	IFNL4
chr19	39246314	39248856	-	ENST00000606380	ENSG00000272395	IFNL4
chr19	39246314	39248856	-	ENST00000616270	ENSG00000272395	IFNL4
chr19	39246314	39248856	-	ENST00000610963	ENSG00000272395	IFNL4
chr19	39264382	39265817	+	ENST00000607083	ENSG00000272311	IFNL4P1
chr19	40222208	40226690	-	ENST00000430325	ENSG00000105219	CNTD2
chr19	40222215	40226657	-	ENST00000221818	ENSG00000105219	CNTD2
chr19	40512614	40575569	+	ENST00000597389	ENSG00000160460	SPTBN4
chr19	40875798	40876747	-	ENST00000602008	ENSG00000198077	CYP2A7
chr19	45769720	45779619	-	ENST00000596686	ENSG00000104936	DMPK
chr19	45778903	45779569	-	ENST00000599392	ENSG00000104936	DMPK
chr19	47432407	47447622	-	ENST00000601757	ENSG00000118160	SLC8A2
chr19	47520690	47545300	-	ENST00000263351	ENSG00000118156	ZNF541

chr19	47521224	47545261	-	ENST00000595558	ENSG00000118156	ZNF541
chr19	48845551	48859382	-	ENST00000595867	ENSG00000105559	PLEKHA4
chr19	48852238	48859374	-	ENST00000594195	ENSG00000105559	PLEKHA4
chr19	48858861	48860118	-	ENST00000596713	ENSG00000105559	PLEKHA4
chr19	49067418	49072941	-	ENST00000221444	ENSG00000104848	KCNA7
chr19	50621979	50668394	-	ENST00000544769	ENSG00000213023	SYT3
chr19	52970445	53037898	-	ENST00000600425	ENSG00000242779	ZNF702P
chr19	52978900	53037883	-	ENST00000594516	ENSG00000242779	ZNF702P
chr22	12602466	12626642	+	ENST00000634617	ENSG00000283023	FRG1GP
chr22	20667836	20670984	-	ENST00000411545	ENSG00000229107	ABHD17AP4
chr22	38085165	38088938	-	ENST00000428572	ENSG00000128298	BAIAP2L2
chr22	38090127	38091559	+	ENST00000609162	ENSG00000272720	CTA-228A9.3
chr22	38213535	38216505	+	ENST00000407965	ENSG00000185022	MAFF
chr22	39994949	40043529	+	ENST00000333407	ENSG00000133477	FAM83F
chr22	39995362	39999125	+	ENST00000488874	ENSG00000133477	FAM83F
chr22	43197283	43343388	-	ENST00000360835	ENSG00000159307	SCUBE1
chr22	43203992	43343283	-	ENST00000615096	ENSG00000159307	SCUBE1
chr22	43237576	43343323	-	ENST00000290460	ENSG00000159307	SCUBE1
chr22	50287987	50301462	-	ENST00000432455	ENSG00000196576	PLXNB2
chr21	7816675	7829926	-	ENST00000622690	ENSG00000276289	KCNE1B
chr21	7816678	7829592	-	ENST00000618699	ENSG00000276289	KCNE1B
chr21	7819044	7829587	-	ENST00000623803	ENSG00000276289	KCNE1B
chr21	10328411	10342669	-	ENST00000622592	ENSG00000277693	AP003900.6
chr21	10328936	10342737	-	ENST00000616952	ENSG00000277693	AP003900.6
chr21	34446688	34459604	-	ENST00000432085	ENSG00000180509	KCNE1
chr21	34446688	34459938	-	ENST00000621601	ENSG00000180509	KCNE1
chr21	34446690	34459604	-	ENST00000399289	ENSG00000180509	KCNE1
chr21	38863676	38912921	-	ENST00000626259	ENSG00000205622	AF064858.6
chr21	45759804	45763758	-	ENST00000617854	ENSG00000275139	RP1-101D8.1

Supplementary Table 5. Promoters specifically marked with H4K3me3 in higher grade samples.

chr	transcript_start	transcript_end	strand	transcript_id	gene_id	gene_name
chr1	9672426	9687555	-	ENST00000415330	ENSG00000231789	PIK3CD-AS2
chr1	110593580	110607429	-	ENST00000633222	ENSG00000177301	KCNA2
chr1	110602998	110607850	-	ENST00000485317	ENSG00000177301	KCNA2
chr1	110603254	110606353	-	ENST00000316361	ENSG00000177301	KCNA2
chr1	110603996	110606364	-	ENST00000525120	ENSG00000177301	KCNA2
chr1	197915837	197932266	+	ENST00000561173	ENSG00000143355	LHX9
chr2	60451167	60553499	-	ENST00000356842	ENSG00000119866	BCL11A
chr2	60451167	60553567	-	ENST00000359629	ENSG00000119866	BCL11A
chr2	60451781	60550914	-	ENST00000489516	ENSG00000119866	BCL11A
chr2	60452500	60553507	-	ENST00000631857	ENSG00000119866	BCL11A
chr2	60457198	60553498	-	ENST00000335712	ENSG00000119866	BCL11A
chr2	60460274	60553431	-	ENST00000358510	ENSG00000119866	BCL11A
chr2	60541785	60553500	-	ENST00000409351	ENSG00000119866	BCL11A
chr3	62369681	62374324	-	ENST00000486811	ENSG00000153266	FEZF2
chr4	2059512	2069088	+	ENST00000423729	ENSG00000185818	NAT8L
chr4	2059812	2069089	+	ENST00000331662	ENSG00000185818	NAT8L
chr4	39498755	39527618	-	ENST00000316423	ENSG00000109814	UGDH
chr4	39498763	39527437	-	ENST00000501493	ENSG00000109814	UGDH
chr4	39498788	39526557	-	ENST00000506179	ENSG00000109814	UGDH
chr4	39499911	39527439	-	ENST00000507089	ENSG00000109814	UGDH
chr4	39510511	39527564	-	ENST00000515021	ENSG00000109814	UGDH
chr4	39510667	39527900	-	ENST00000514106	ENSG00000109814	UGDH
chr4	39510697	39527915	-	ENST00000510881	ENSG00000109814	UGDH
chr4	39510709	39527437	-	ENST00000503779	ENSG00000109814	UGDH
chr4	39510736	39527437	-	ENST00000509391	ENSG00000109814	UGDH
chr4	39510815	39528233	-	ENST00000515398	ENSG00000109814	UGDH
chr4	39514093	39528311	-	ENST00000505698	ENSG00000109814	UGDH
chr4	39521352	39527598	-	ENST00000510490	ENSG00000109814	UGDH
chr4	39528019	39594707	+	ENST00000504032	ENSG00000249348	UGDH-AS1
chr5	141484997	141487789	+	ENST00000617094	ENSG00000242419	PCDHGC4
chr5	141485063	141485767	+	ENST00000610539	ENSG00000242419	PCDHGC4
chr5	141485072	141511496	+	ENST00000618371	ENSG00000242419	PCDHGC4
chr5	141485174	141512979	+	ENST00000306593	ENSG00000242419	PCDHGC4
chr6	81491439	81751812	-	ENST00000412306	ENSG00000112773	FAM46A
chr6	81745730	81752708	-	ENST00000369754	ENSG00000112773	FAM46A
chr6	81745730	81752708	-	ENST00000320172	ENSG00000112773	FAM46A
chr6	81745730	81752774	-	ENST00000369756	ENSG00000112773	FAM46A
chr6	81749870	81751753	-	ENST00000423467	ENSG00000112773	FAM46A
chr7	55109723	55146724	+	ENST00000450046	ENSG00000146648	EGFR
chr7	151081080	151083546	-	ENST00000297533	ENSG00000164897	TMUB1
chr7	151081484	151083400	-	ENST00000482202	ENSG00000164897	TMUB1
chr7	151081496	151083232	-	ENST00000476627	ENSG00000164897	TMUB1
chr8	27597808	27611431	-	ENST00000405140	ENSG00000120885	CLU
chr8	27597996	27611357	-	ENST00000523500	ENSG00000120885	CLU
chr8	27604366	27611751	-	ENST00000560566	ENSG00000120885	CLU
chr8	27606354	27611966	-	ENST00000519742	ENSG00000120885	CLU
chr9	136006537	136050580	-	ENST00000371753	ENSG00000148411	NACC2

chr11	57460549	57477534	+	ENST00000335099	ENSG00000186907	RTN4RL2
chr11	57460857	57468884	+	ENST00000533205	ENSG00000186907	RTN4RL2
chr11	57460866	57468630	+	ENST00000395120	ENSG00000186907	RTN4RL2
chr10	28677342	28682939	+	ENST00000375533	ENSG00000095739	BAMBI
chr10	28677510	28682677	+	ENST00000497699	ENSG00000095739	BAMBI
chr12	57096341	57129100	-	ENST00000556155	ENSG00000166888	STAT6
chr12	57128493	57150059	+	ENST00000553277	ENSG00000123384	LRP1
chr12	57128499	57213351	+	ENST00000243077	ENSG00000123384	LRP1
chr12	57128716	57150059	+	ENST00000338962	ENSG00000123384	LRP1
chr12	57128909	57155593	+	ENST00000554174	ENSG00000123384	LRP1
chr16	30085793	30091884	-	ENST00000395224	ENSG00000149922	TBX6
chr16	30085796	30091249	-	ENST00000279386	ENSG00000149922	TBX6
chr16	30085796	30091249	-	ENST00000627355	ENSG00000149922	TBX6
chr16	30086035	30091213	-	ENST00000567664	ENSG00000149922	TBX6
chr16	30087916	30091887	-	ENST00000553607	ENSG00000149922	TBX6
chr19	50311942	50333515	-	ENST00000474951	ENSG00000131398	KCNC3
chr19	50333796	50334561	-	ENST00000531692	ENSG00000131401	NAPSB

Supplementary Table 6. KEY RESOURCES TABLE

REAGENT or RESOURCE	SOURCE	IDENTIFIER
**Antibodies**		
Rabbit polyclonal anti-H3K4me3	Merck Millipore	Cat. number 07-473, RRID:AB_1977252
Rabbit polyclonal anti-H3K27ac	Active Motif	Cat. number 39133, RRID:AB_2561016
Rabbit monoclonal anti-H3K27me3	Abcam	Cat. number ab192985, RRID:AB_2650559
Rabbit polyclonal anti-FOXM1	Diagenode	Cat. number C15410232-100
Rabbit normal IgG antibody	Merck Millipore	PP64B
Rabbit Normal IgG Control Antibody, Unconjugated	Cell Signalling	Cat. number 2729, RRID:AB_1031062
**Biological Samples**		
See Table S1	This paper	NA
**Chemicals, Peptides, and Recombinant Proteins**		
37% Formaldehyde	Sigma-Aldrich	CAS number 50-00-0
A- and G-Sepharose beads	Merck Millipore	Cat. number 16-156 & 16-266
Adapters and USER enzyme	New England Biolabs	Cat. number E7335L
Ampure Beads	Beckman Coulter	Cat. number A63880
DMEM	ThermoFisher Scientific	Ref 31885-023
DMEM/F-12, GlutaMAX™	ThermoFisher Scientific	Ref 31966-021
DNA polymerase	EURx	Cat. number E2500-01
Dynabeads Protein A	ThermoFisher Scientific	Cat. number 10002D
FBS	ThermoFisher Scientific	Cat. number 1056000
MboI	NEB	Cat. number R0147M
Proteinaise K	Applied Biosystems	Cat. number 4333793
RNase A	Invitrogen	Cat. number 12091-021
SimplySafe™	EURx	Cat. number E4600-01
SYBR Green chemistry	Applied Biosystem by Thermo Fisher Scientific	Cat. Number 4385612
T4 DNA ligase	NEB	Cat. number M0202T
TRI Reagent	Sigma-Aldrich	Product No. T9424
**Critical Commercial Assays**		
Agilent DNA High Sensitivity Kit	Agilent Technologies, Ltd.	Cat. number 5067-4626
Agilent RNA 6000 Nano Kit	Agilent Technologies, Ltd.	Cat. number 5067-1511
ChIP-IT Express Immunoprecipitation Kit	Active Motif	Cat. number 53008
EZ-96 DNA Methylation-Gold kit	Zymo Research	Cat. number D5007
Infinium HumanMethylation 450 BeadChip	Illumina	Cat. IDs: WG-314-1003
KAPA Stranded mRNA Sample Preparation Kit	Kapa Biosystems	Kit code KK8420 Roche cat no. 07962193001
NEBNext Ultra Library Prep Kit for Illumina	Ilumina	Cat. number E7370S/L
Nextera DNA Library Preparation Kit	Illumina	Cat. number FC-121-1030
Syngen GEL/ PCR Mini Kit	Syngen Biotech	Cat. number SY201010
QIAsymphony DNA Midi kit	Qiagen	Cat. number 931255

QuantiFluor double stranded DNA System	Promega	Cat. number E2670
RNeasy Mini kit	(Qiagen, Hilden, Germany)	Cat. Number 74104
xGen Lockdown Probes and Reagents kit	IDT	Cat. number 1072280
Ideal ChIP-seq Kit for Transcription Factors	Diagenode	C01010055
Zymo DNA Clean and Concentrator 5	Zymo Research	Cat. number D4003T
**Deposited Data**		
DNA methylation data from human brain cerebellum and cortex	[1]	GSE61431
DNA methylation data from pilocytic astrocytoma samples	[2]	GSE44684
Gene expression data from pilocytic astrocytoma samples	[2]	GSE44971
**Experimental Models: Cell Lines**		
LN229	ATCC	CRL-2611
LN18	ATCC	CRL-2610
U87	ATCC	HTB-14
WG4	Bozena Kaminska Lab	NA
IPIN	Bozena Kaminska Lab	NA
**Oligonucleotides**		
IDH_mut_F GGATGCTGCAGAAGCTATAA	Oligo.pl	NA
IDH_mut_R CATGCAAAATCACATTATTGCC	Oligo.pl	NA
GAPDH_ChIP_F TACTAGCGGTTTTACGGGCGCAC	Oligo.pl	NA
GAPDH_ChIP_R TCGAACAGGAGGAGCAGAGAGCGA	Oligo.pl	NA
HOXA7_ChIP_F AGATGCGGAAATTGGCCTCAG	Oligo.pl	NA
HOXA7_ChIP_R TCCTACGACCAAAACATCCCC	Oligo.pl	NA
CCNB1_prom_ChIP_F CGCGATCGCCCTGGAAACGCA	Oligo.pl	NA
CCNB1_prom_ChIP_R CCCAGCAGAAACCAACAGCCGT	Oligo.pl	NA
ANXA2R_enh_ChIP_F ATGGACAAACAAACCAACAAACA	Oligo.pl	NA
ANXA2R_enh_ChIP_R AGTGCAGTCCATGCAGGTTA	Oligo.pl	NA
ANXA2R_qPCR_F CAAGTACAGCGAAGCCCACT	Oligo.pl	NA
ANXA2R_qPCR R CTGAGTCTGTCGGGTTCCTC	Oligo.pl	NA
FOXM1_qPCR_F GCAGCGACAGGTTAAGGTTG	Oligo.pl	NA
ANXA2R_enh_set1_F	Oligo.pl	TCTGCTAGAGATGTTTGCCTTTCT
ANXA2R_enh_set2_F	Oligo.pl	TAGATCTGCTAGAGATGTTTGCC
ANXA2R_enh_R (paired with both _set1_F and _set2_F)	Oligo.pl	CAGTCCATGCAGGTTACTTCTTG

FOXM1_qPCR_R GTCATGCGCTTCCTCTCAGT	Oligo.pl	NA
H19 imprinting primer pair	Diagenode	C01010055
Myoglobin primer pair	Diagenode	C01010055
CCND1 primer pair	Oligo.pl	Sanders et al., 2013
CCNB1 primer pair	Oligo.pl	Sanders et al., 2013
PLK1 primer pair	Oligo.pl	Sanders et al., 2013
**Software and Algorithms**		
Basic4CSeq Biocoductor package	3	
bedtools	4	
bigWigAverageOverBed		https://github.com/ENCODE-DCC/kentUtils
Bowtie 2.2.6.2	5	
BSMAP	6	
CytoMeth	Draminski et al. in prep.	https://github.com/mdraminski/CytoMeth
Dendextend R package	7	
EDASeq	8	
FastQC		https://www.bioinformatics.babraham.ac.uk/projects/fastqc/
FASTQ Trimmer	9	
FASTX		http://hannonlab.cshl.edu/fastx_toolkit/
Filter FASTQ	9	
F-seq1.85	10	
HiCEnterprise	11	https://github.com/hansiu/HiCEnterprise
HiTC Bioconductor package	12	
MACS2.1	13	
methylKit	14	
PANTHER Overrepresentation Test (Released 20171205)	15	
Picard		http://broadinstitute.github.io/picard/
PWMEnrich R package	16	
QuasR Bioconductor package	17	
samtools	18	
Tophat2	19	
Trimmomatic	20	
**Other**		
ENCODE Regulation 'Txn Factor' track data V3		http://hgdownload.soe.ucsc.edu/goldenPath/hg19/encodeDCC/wgEncodeRegTfbsClustered/
GO Ontology database Released 2018-06-01	21,22	
HOCOMOCO v11 database	23	
PhastCons 100-Way scores		http://hgdownload.cse.ucsc.edu/goldenPath/hg38/phastCons100way/hg38.phastCons100way.bw

Supplementary References

1. Pidsley, R. *et al.* Methylomic profiling of human brain tissue supports a neurodevelopmental origin for schizophrenia. *Genome Biol.* (2014). doi:10.1186/s13059-014-0483-2
2. Lambert, S. R. *et al.* Differential expression and methylation of brain developmental genes define location-specific subsets of pilocytic astrocytoma. *Acta Neuropathol.* **126**, 291–301 (2013).
3. Walter, C., Schuetzmann, D., Rosenbauer, F. & Dugas, M. Basic4Cseq: an R/Bioconductor package for analyzing 4C-seq data. *Bioinformatics* **30**, 3268–3269 (2014).
4. Quinlan, A. R. & Hall, I. M. BEDTools: A flexible suite of utilities for comparing genomic features. *Bioinformatics* (2010). doi:10.1093/bioinformatics/btq033
5. Langmead, B. & Salzberg, S. L. Fast gapped-read alignment with Bowtie 2. *Nat. Methods* **9**, 357–359 (2012).
6. Xi, Y. & Li, W. BSMAP: Whole genome bisulfite sequence MAPping program. *BMC Bioinformatics* (2009). doi:10.1186/1471-2105-10-232
7. Galili, T. dendextend: An R package for visualizing, adjusting and comparing trees of hierarchical clustering. *Bioinformatics* (2015). doi:10.1093/bioinformatics/btv428
8. Risso, D. EDASeq: Exploratory Data Analysis and Normalization for RNA-Seq. (2013). doi:10.18129/B9.bioc.EDASeq
9. Blankenberg, D. *et al.* Manipulation of FASTQ data with galaxy. *Bioinformatics* (2010). doi:10.1093/bioinformatics/btq281
10. Boyle, A. P., Guinney, J., Crawford, G. E. & Furey, T. S. F-Seq: A feature density estimator for high-throughput sequence tags. *Bioinformatics* (2008). doi:10.1093/bioinformatics/btn480
11. Kranas, H., Tuszyńska, I. & Wilczynski, B. HiCEnterprise: Identifying long range chromosomal contacts in HiC data. (2019). doi:https://doi.org/10.7287/peerj.preprints.27753v1
12. Servant, N. *et al.* HiTC: Exploration of high-throughput 'C' experiments. *Bioinformatics* (2012). doi:10.1093/bioinformatics/bts521
13. Zhang, Y. *et al.* Model-based analysis of ChIP-Seq (MACS). *Genome Biol.* (2008). doi:10.1186/gb-2008-9-9-r137
14. Akalin, A. *et al.* MethylKit: a comprehensive R package for the analysis of genome-wide DNA methylation profiles. *Genome Biol.* (2012). doi:10.1186/gb-2012-13-10-R87
15. Mi, H. *et al.* PANTHER version 11: Expanded annotation data from Gene Ontology and Reactome pathways, and data analysis tool enhancements. *Nucleic Acids Res.* (2017). doi:10.1093/nar/gkw1138
16. Stojnic, R. & Diez, D. PWMEnrich: PWM enrichment analysis. (2018).
17. Gaidatzis, D., Lerch, A., Hahne, F. & Stadler, M. B. QuasR: Quantification and annotation of short reads in R. *Bioinformatics* (2015). doi:10.1093/bioinformatics/btu781
18. Li, H. *et al.* The Sequence Alignment/Map format and SAMtools. *Bioinformatics* (2009). doi:10.1093/bioinformatics/btp352
19. Kim, D. *et al.* TopHat2: accurate alignment of transcriptomes in the presence of insertions, deletions and gene fusions. *Genome Biol.* (2013). doi:10.1186/gb-2013-14-4-r36
20. Bolger, A. M., Lohse, M. & Usadel, B. Trimmomatic: A flexible trimmer for Illumina sequence data. *Bioinformatics* (2014). doi:10.1093/bioinformatics/btu170
21. Ashburner, M. *et al.* Gene ontology: Tool for the unification of biology. *Nature Genetics* (2000). doi:10.1038/75556
22. Carbon, S. *et al.* Expansion of the gene ontology knowledgebase and resources: The gene ontology consortium. *Nucleic Acids Res.* (2017). doi:10.1093/nar/gkw1108
23. Kulakovskiy, I. V. *et al.* HOCOMOCO: Towards a complete collection of transcription factor binding models for human and mouse via large-scale ChIP-Seq analysis. *Nucleic Acids Res.* (2018). doi:10.1093/nar/gkx1106
